# Birds in the playground: Evaluating the effectiveness of an urban environmental education project in enhancing school children’s awareness, knowledge and attitudes towards local wildlife

**DOI:** 10.1371/journal.pone.0193993

**Published:** 2018-03-06

**Authors:** Rachel L. White, Katie Eberstein, Dawn M. Scott

**Affiliations:** 1 School of Pharmacy and Biomolecular Sciences, University of Brighton, Brighton, United Kingdom; 2 Brighton and Hove environmental education, Sussex Wildlife Trust, Brighton, United Kingdom; Waseda University, JAPAN

## Abstract

Children nowadays, particularly in urban areas, are more disconnected from nature than ever before, leading to a large-scale “extinction of experience” with the natural world. Yet there are many potential benefits from children interacting with nature first-hand, including via outdoor learning opportunities. Urban environmental education programmes typically aim to increase awareness and knowledge of local biodiversity and to promote positive attitudes and behaviour towards the environment. However, limited research has been conducted evaluating to what extent these interventions achieve their goals. Here, we explore and assess the influence of a six-week bird-feeding and monitoring project conducted within school grounds (“Bird Buddies”) on individual awareness, knowledge and attitudes towards birds by primary school children. This initiative was conducted across eight (sub-)urban primary schools within Brighton and Hove (UK), with 220 participating children (aged 7 to 10). Via pre- and post-project questionnaires, we found evidence for enhanced awareness of local biodiversity, alongside significant gains in both bird identification knowledge and attitudes, which were greatest for children with little prior exposure to nature. Many children expressed a keenness to continue improving the environmental value of their school grounds and to apply elements of the project at home. Student project evaluation scores were consistently positive. Mirroring this, participating teachers endorsed the project as a positive learning experience for their students. One year after the project, several schools were continuing to feed and watch birds. Collectively, the findings from this study highlight the multiple benefits that can be derived from engagement with a relatively short outdoor environmental activity. We therefore believe that such interventions, if repeated locally/longer term, could enhance children’s experience with nature in urban settings with combined positive environmental impact.

## Introduction

Worldwide, children are becoming less likely to have direct contact with nature [1]. A key driver is rapid and ongoing urbanisation, which can result in a marked reduction in opportunities to experience nature, as a high proportion of urban areas are composed of artificial materials and segregated from natural systems and processes [2–8]. Additionally, the continuing growth in sedentary pastimes (e.g. watching TV, playing computer games, and using the Internet) has decreased children’s time available for engaging in such interaction [9–10]. Consequently, many children are highly disconnected from nature [1,11], and this ongoing alienation has been labelled “the extinction of experience” [12–13]. This phenomenon has not only been shown to diminish a wide range of health and wellbeing benefits for people [14–16], but also a decline in emotional affinity towards nature and both pro-environmental attitudes and behaviour (reviewed in [1]). Therefore, how best to reconnect and engage people with nature is a fundamental research priority [1,11]–especially concerning children, as evidence suggests childhood nature experiences can positively affect adult environmental attitudes and behaviour [17–22].

Environmental knowledge can facilitate attitude formation [23–24], and although the link between knowledge and attitudes is not always clear, both ultimately can influence environmental behaviours [25]. It is important to emphasise that environmental knowledge is multifaceted, with species identification recognised as a fundamental component (see [26]). Loss of familiarity with the natural world, particularly in Western countries, is resulting in a loss of environmental knowledge–including the ability to identify even the most common species [11,26]. Possessing at least basic animal and plant identification skills is often emphasised as a prerequisite for understanding and appreciating biodiversity, because the species is the fundamental unit of biodiversity (see [27–28]). Few studies have examined children’s prevailing level of species identification skills, but generally find poor identification ability for common native species, even for birds despite having the advantage of being relatively conspicuous, charismatic, well-described and provisioned with field guides (e.g. [9,29–32]). Factors studied with respect to children’s ability to identify species include gender and age [29,31–36], pet ownership [34,37], experiences with nature [38], native versus exotic species [9,29,31], educational institution [39], and the medium with which the specimens are presented [32,35,40–41]. However, specific trends are difficult to establish due to the limited number of studies, conflicting findings, and a lack of analyses exploring the relative importance of different factors (i.e. multivariate techniques). Although not explicitly studied, a likely contributing factor to poor identification knowledge is the increasing neglect of taxonomic education within schools [40,42].

Conceptualising attitudes is a complex task, and there are various ways to define and assess attitudes. Serpell [43] suggests there are two primary motivational determinants of attitude, labelled ‘affect’ (the affective and/or emotional response) and ‘utility’ (perceptions of instrumental value), which are typically represented as a continuum between positive and negative poles on a two-dimensional axis. As with environmental knowledge (and its components), numerous factors can affect children’s attitudes towards wildlife, including: gender [8,33–34,44–48], age [33–34,44,48], pet ownership [34,37,44,49–50], experiences with nature [6,8,33,44,46,49], and species [49–51]. Few studies have explicitly investigated the interrelationship between children’s knowledge of and attitudes towards wildlife, and with mixed findings. For example, positive [34], negative [52] and no [53] relationship between knowledge of and attitudes towards birds have been reported.

Environmental education is defined as “a learning process in which individuals gain awareness of their environment, acquire knowledge, skills, values and experiences, which will subsequently enable them to act—individually and collectively—to solve environmental problems” [54]. Incorporating environmental education opportunities within school curricula is one tool that can potentially assist with reversal of the extinction of experience.

Although an increasing number of environmental education programmes exist, scientifically robust evaluations of their effectiveness at meeting learning outcomes remain scarce [55]. This is at least partly due to many studies focusing on assessing short-term changes only (i.e. immediately after participating) and neglecting their longer-term impact [56]. Nevertheless, programmes that incorporate action-based outdoor learning are widely believed to be particularly beneficial [57–58]. Given that outdoor learning in distant settings is becoming increasingly difficult for many schools and that experiences with nature are not limited to engagement with pristine or wilderness environments, urban green-spaces near schools and school grounds themselves can offer a viable alternative [59–60]. Indeed, school grounds are arguably the main, and perhaps only, area where urban children can have sustained and regular direct contact with local nature, in turn facilitating engagement and learning outcomes [58].

To date, outdoor school-based environmental education has typically focused on school ground improvement and greening initiatives, horticultural projects, and outdoor play developments, but have only recently become the focus of empirical inquiry [60]. Such studies are biased towards gardening projects within the USA where, although some initiatives have improved environmental knowledge and attitudes (e.g. [61–63]), collectively their findings are ambiguous [64]. Comparatively few initiatives and evaluative studies have investigated environmental education centred on urban biodiversity (specifically animals), despite its potential importance being highlighted [65].

Wild birds likely form the main, or at least most readily recognised, wildlife interaction that people living in urban areas can experience in daily life [65]. It is therefore surprising that there is a general lack of environmental education projects focused on urban birds. Both Brossard et al. [66] and Evans et al. [67] report the findings of two USA programmes where adult participants established and/or monitored bird nesting sites at home. Although some evidence was found for an increase in avian knowledge, findings were inconsistent regarding change in environmental attitudes. Bogner [68] examined a largely classroom-based extra-curricular education unit for Swiss school children focused on the common swift (*Apus apus*), and found a positive effect on knowledge about the species with an increase in environmental attitudinal score for two of the five studied dimensions (“enjoyment of nature” and “intent of support”). In addition, although not focused on birds, the Swiss-based educational programme “Nature on the Way to School”, which included a wide-range of exploratory learning activities about local nature, found participation significantly increased the number and diversity of species children noticed walking to school [69–70].

Bird feeding is widespread in Western countries and growing in popularity [71], and is increasingly recognised as being a potentially important, yet understudied, tool for stimulating a broader interest in the natural world [53,72]. A home-based programme in the USA encouraged children to learn about and feed birds in their garden [53]. The programme found some evidence of an increase in avian knowledge but no change in environmental attitudes, but Beck et al. [53] called for further research. Although a handful of initiatives do exist which encourage children to survey wildlife in their school grounds (e.g. RSPB’s Big Schools’ Birdwatch www.rspb.org.uk/schoolswatch/), to the best of our knowledge, no study has focused on and assessed the value of children feeding and monitoring (i.e. regular repeat engagement) local wildlife in such a setting.

Here, we evaluate the effectiveness of an urban school-based environmental education project to improve children’s awareness, environmental knowledge (i.e. species identification) and attitudes towards local wildlife, via active participation in bird feeding and monitoring. Pre- and post-project questionnaires were used to assess baseline and change in knowledge and attitudes, their interrelationship, and the influence of sociodemographic variables. Enjoyment and short-term benefits were directly assessed via student and teacher feedback. Finally, a one-year follow-up with teachers assessed continued interest in birds and bird feeding at the class/school level, alongside collating evidence of long-term benefits and wider impact.

## Materials and methods

### “Bird Buddies” programme

“Bird Buddies” is a UK-based environmental education project designed for urban primary school children aged 7–10. The main goals of the project were to provide children with opportunities to experience nature first-hand, learn about and value local biodiversity, undertake wildlife monitoring, and show how they can make positive changes to their environment and attract wildlife. Birds are an ideal wildlife group to study in this capacity as they are easily seen, identified and can respond quickly to resource enhancement [73]. For each participating class, the project comprised of a six-week bird-feeding and monitoring initiative within their school grounds. Firstly, a project introduction and engagement workshop was run per class, which included interactive hands-on activities focusing on bird identification and ecology. Secondly, two weeks of surveying without bird feeding was conducted followed by four weeks of surveying with bird feeding. All necessary bird feeding and surveying equipment were provided for the participating schools. See S1 Appendix for further details regarding the “Bird Buddies” workshop, bird feeding and monitoring protocols.

### Study area, school selection and participants

The “Bird Buddies” programme was conducted at eight primary schools within the city of Brighton and Hove (East Sussex, UK) during April and May 2016. The city covers approximately 88 km^2^ with some 275,800 inhabitants and a population density of 33.1 persons per hectare [74]. It is bounded by green and open space, namely the South Downs National Park and the seafront, and is part of the Brighton & Lewes Downs UNESCO World Biosphere Region. School grounds constitute 15% of the administrative area of Brighton and Hove [75]. Schools were recruited through online advertisements and a presentation at an event aimed at local teachers. Selected schools had to meet three criteria: 1) (sub)-urban in locality; 2) have not previously monitored or undertaken supplemental feeding of birds in their school grounds, and 3) able to fully commit to the project for the duration. Each participating school nominated one class to take part in the project.

### Pre-project student questionnaire

We developed a structured questionnaire to assess participating children’s pre-project (i.e. base-line) experience with nature, and knowledge of and attitudes towards common UK garden birds. The questionnaire was piloted with several similarly aged children from non-participating schools to check for clarity. One week prior to the project workshop, paper copies of the questionnaire were administered to participants by their teacher during class. The teachers had been instructed to provide no contextual information about the project or reason for completing the questionnaire, and to introduce it as a survey and not as a test or exam. The questionnaire was completed independently by the participants in class, under supervised conditions. Help could be asked for with reading and explaining the questions only. The questionnaires were filled in anonymously and typically completed within 15 minutes.

The questionnaire comprised of 10 items across three sections. Section one (sociodemographic status) focused on the participant’s personal background and experience with nature, specifically: gender, presence of outside space at home, whether or not they see birds and/or if their family feed birds in their outside space at home, pet ownership, and what different nature activities (both direct and vicarious; n = 10) they have participated in over the past year. The second section related to attitudes towards birds, including using a five-point Likert scale to rank how they feel about birds (5 = “I really like birds” to 1 = “I really don’t like birds”) and, if they have been bird-watching before, how did they rate the experience (5 = “It was really fun” to 1 = “I really didn’t like it”). They were also asked to answer either “yes” or “no” to eight questions–four relating to the ‘utility’ of birds and four concerning affection/emotion (‘affect’) towards birds. The final section focused on bird identification and asked them to identify 12 species of bird from pictures, namely: Blue tit (*Cyanistes caeruleus*), Robin (*Erithacus rubecula*), House sparrow (*Passer domesticus*), Chaffinch (*Fringilla coelebs*), Greenfinch (*Carduelis chloris*), Wren (*Troglodytes troglodytes*), Blackbird (*Turdus merula*), Starling (*Sturnus vulgaris*), Magpie (*Pica pica*), Collared dove (*Streptopelia decaocto*), Carrion crow (*Corvus corone*) and Black-headed gull (*Chroicocephalus ridibundus*). These species were selected as they are all common UK garden birds and consistently listed within the top sightings of the RSPB’s Big Schools’ Birdwatch. The illustrations featured a colour drawing of an adult male bird of each species (not to scale). See S2 Appendix for the complete pre-project questionnaire and associated coding.

### Post-project student questionnaire

To measure changes in knowledge of and attitudes towards birds, participants completed a questionnaire at the end of the project, similar in content and delivery as the pre-project questionnaire. They were first asked to use a five-point Likert scale to rank how they felt about birds, and, if they had been bird-watching outside of school since the start of the project, how they rated the experience. Participants also answered again the attitudinal questions towards birds, along with the bird identification test. The final section of the questionnaire involved both closed and open project evaluation questions. Part of this evaluation comprised of nine statements regarding their attitudes towards specific elements of the project using a five-point Likert scale (5 = “strongly agree” to 1 = “strongly disagree”) and summed to provide a student evaluation score (min. = 9, max. = 45), with high internal reliability (Cronbach’s α = 0.85). See S2 Appendix for the complete post-project questionnaire and associated coding.

### Teacher project evaluation and long-term follow-up

Immediately after the project, the lead teachers were emailed a nine-item, semi-structured project evaluation questionnaire (see S3 Appendix for complete version). To summarise, the seven open-answered questions asked them to explain their motivation for involvement, comment on the student’s enjoyment, and the anticipated long-term benefits. They were asked to identify and explain what they liked least and most about the project, provide any recommendations for improvement, and register their interest in participating in a similar project in the future. Additionally, the teachers were asked to rate various aspects of the project using a five-point Likert scale (13 statements, 5 = “strongly agree” to 1 = “strongly disagree” or 5 = “Excellent” to 1 = “Poor”). One statement “The project was time consuming” was reverse-scored. A teacher evaluation score (min. = 13, max. = 65) was created by summing these individual statement ratings (Cronbach’s α = 0.74).

One year after the project’s end, a short online questionnaire (see S3 Appendix) was sent to each participating teacher to determine if the class/school were still feeding and or surveying birds and if so, the extent and type of bird feeding/surveying activity. They were also asked if the “Bird Buddies” initiative had encouraged the class/school to participate in other environmental activities.

### Data analysis

All raw data from the four questionnaire types (pre-project, post-project, teacher project evaluation and long-term follow-up) were checked for errors prior to analysis and anomalies removed. The coding of the answers follows that shown in S2 and S3 Appendices. Descriptive statistics (i.e. average, standard deviation, frequency, proportions) were first derived and examined for each questionnaire item and sociodemographic predictor (seven factors: school, class year, gender, outdoor space at home, birds seen at home, feed birds at home, and pet ownership; one continuous variable: number of nature activities participated in). Subsequent statistical analyses were performed using R 3.2.5 [76].

For the bird identification test in both the pre- and post-project questionnaires, identification was determined as either correct (2), partially correct (1) or incorrect (0). To score two points, the full common name had to be given; e.g. blue tit or black-headed gull. If only the common genus or family name was correct then it scored 1 (e.g. tit instead of blue tit or herring gull *Larus argentatus* instead of black-headed gull), otherwise the value 0 was assigned. Spelling was not penalised as long as the common name could be determined. For each student, both a pre- and post-project identification score was calculated as the number of species correctly identified (12 species, maximum score of 24), and both found to be not normally distributed. Change in identification score (normally distributed) was calculated as the difference between scores and used as a measure of learning effect. All incorrect names were noted along with whether or not they referred to either a non-native or “made-up” species. We calculated the proportion of children within each year group and school that could correctly identify each species and the overall proportion of correct identifications for the species as a whole. Pre- and post-project bird identification scores were compared via paired Wilcoxon signed-rank tests—for all participants collectively and by each sociodemographic factor. Generalised linear mixed models (GLMM) (using the ‘lme4’ package [77]) were constructed to explore the effect of the sociodemographic predictors on children’s pre- and post-project ability to identify birds and change in identification score (response variables). Both pre- and post-project identification scores were entered into their respective models as the number of correct answers minus number of incorrect answers (using the ‘cbind’ function), and a binomial error structure with logit link function used, with school included as a random effect. Generalised Variance Inflation Factors (GVIFs) were used to check for multi-collinearity between predictors and found to be within acceptable norms for all final models, with GVIFs <1.1. First, a global model was built including all sociodemographic variables plus, to control for prior knowledge, pre-project identification score was included as an extra predictor for the post-project model. Minimum adequate models (MAM) were selected based on Akaike Information Criterion (AIC) using a stepwise selection procedure and a 0.05 significance level. R^2^ values were calculated using the method described by Nakagawa & Schielzeth [78]. The same process was used to model change in identification score, but with a Gaussian error structure and identity link function.

For the question “how do you feel about birds?”, pre- and post-project answers were compared via a paired Wilcoxon signed-rank test, and a proportional odds logistic regression (POLR) was used to test for a relationship with pre- and post-project identification scores.

Answers for each affect/utility attitudinal statement were coded either 1 (positive response) or -1 (negative response), as shown in S2 Appendix. For both the pre- and post-project responses, affect and utility scores were calculated per student by summing the responses to the four affect questions and four utility questions separately and then combined to give a composite attitude score (min. = -8 and max. = 8). Composite attitude scores were not normally distributed whereas change in attitude score was. As for bird identification, pre- and post-project attitudinal scores were compared via paired Wilcoxon signed-rank tests. A GLMM was produced for pre-project composite attitude including all sociodemographic variables plus pre-project identification score as predictors and school as a random effect. As the distribution of the response variable was moderately negatively skewed, to improve model fit, a reflected transformation was first applied. Reflection was achieved using the formula (Largest value n_L_ + 1)–(Original value n_x_) and subsequent data points log_10_ transformed and reflected back to ease model interpretation. Change in composite attitude score was modelled with a Gaussian error structure and identity link function. For these two models, simplification and validation checks followed the same process outlined above for the bird identification models. Post-project composite attitude was severely negatively skewed and so could not be modelled in a GLMM framework. Mann-Whitney and Kruskal-Wallis tests were used to identify differences in attitude score (composite, affect and utility) across levels for each sociodemographic factor. To explore pair-wise associations between identification and attitude scores, we performed a series of Kendall’s Tau correlations.

Answers to closed questions in both the student and teacher project evaluations and long-term follow-up questionnaire were explored using descriptive statistics only. Answers to open questions were grouped into themes by hand just from the content itself, and then the frequency of responses falling under each theme were recorded and independently verified.

As discussed by Ballouard et al. [79], written questionnaires administered during class are well adapted to and offer multiple advantages when surveying schoolchildren. However, one potential source of bias is the tendency of respondents to answer questions in a way they deem to be more socially acceptable than would be their “true” answer (i.e. social desirability bias). We reduced the likelihood of its occurrence by asking the teacher’s to assure the student’s that the questionnaires were anonymous and not a formal assessment [79–80].

### Ethics statement

Approval for this study was granted by the University of Brighton’s School of Pharmacy and Bimolecular Sciences Ethics Committee. Permission from schools and parents of all participating school children was obtained. All questionnaire responses were anonymous. Each school was given a unique letter ranging from A to H, and each student assigned a unique number as an identifier in order to match pre- and post-project questionnaires for a given individual.

## Results

A total of 220 children from eight classes across eight different primary schools participated in the “Bird Buddies” project. Specifically, four Year 5 classes (9–10 year olds; n = 108), two Year 4 classes (8–9 year olds; n = 64) and two Year 3 classes (7–8 year olds; n = 48). Average class size was 27.5 children (SD = 5.4) and the sample was slightly male biased (58.6%). Two children missed the pre-project questionnaire and 14 missed the post-project questionnaire–leaving 204 children who attempted both (although not all respondents answered every questionnaire item). See S4 Appendix for full closed-answer pre- and post-project questionnaire results).

Overall, the majority of participants had outside space at home (89.9%), of those 91.8% reported seeing birds within it and 55.6% of participant’s families feed birds either year round or sometimes. Roughly two thirds of participants owned one or more pets (65.1%)—typically a cat or dog. See Table A in S5 Appendix for a full breakdown of the sociodemographic factors. The average number of different nature activities that participants had engaged with over the past year before the project was 4.7 (SD = 2.5; min. = 0, max. = 10). The most participated activities were visiting the countryside/park (81.9%), visiting the zoo (60.8%), and watching wildlife on TV (42.4%). Prior to the project, 159 (72.9%) children had been bird-watching at least once before in some capacity, of which 71.1% found the experience “(really) fun”, although 3.8% “(really) didn’t like it”.

### Bird surveys

Although beyond the scope of this paper, it is important to briefly summarise the key findings from the bird surveys conducted by the classes. Across all participating schools, 21 bird species were observed; however, no change in species richness was found when feeding occurred, for all schools collectively and within individual schools, except for “School H” which saw a significant increase (Mann Whitney: W = 7.0, p < 0.005). A significant difference in species richness was observed across schools for both non-feeding (Kruskal Wallis: x^2^ = 28.8, p < 0.001) and feeding (Kruskal Wallis: x^2^ = 33.8, p < 0.001) surveys. Bird species most seen in the pre-feeding surveys were herring gull, woodpigeon (*Columba palumbus*), carrion crow and robin, whereas the most sighted species during feeding surveys were herring gull, woodpigeon, starling, blackbird and blue tit (see Table B in S5 Appendix for an overview of which species were sighted at least once by each school). Abundance data proved difficult for classes to record—as indicated by frequent use of question marks and/or a range of values. Consequently, we did not attempt to analyse this data.

### Bird identification knowledge

**Fig 1:**
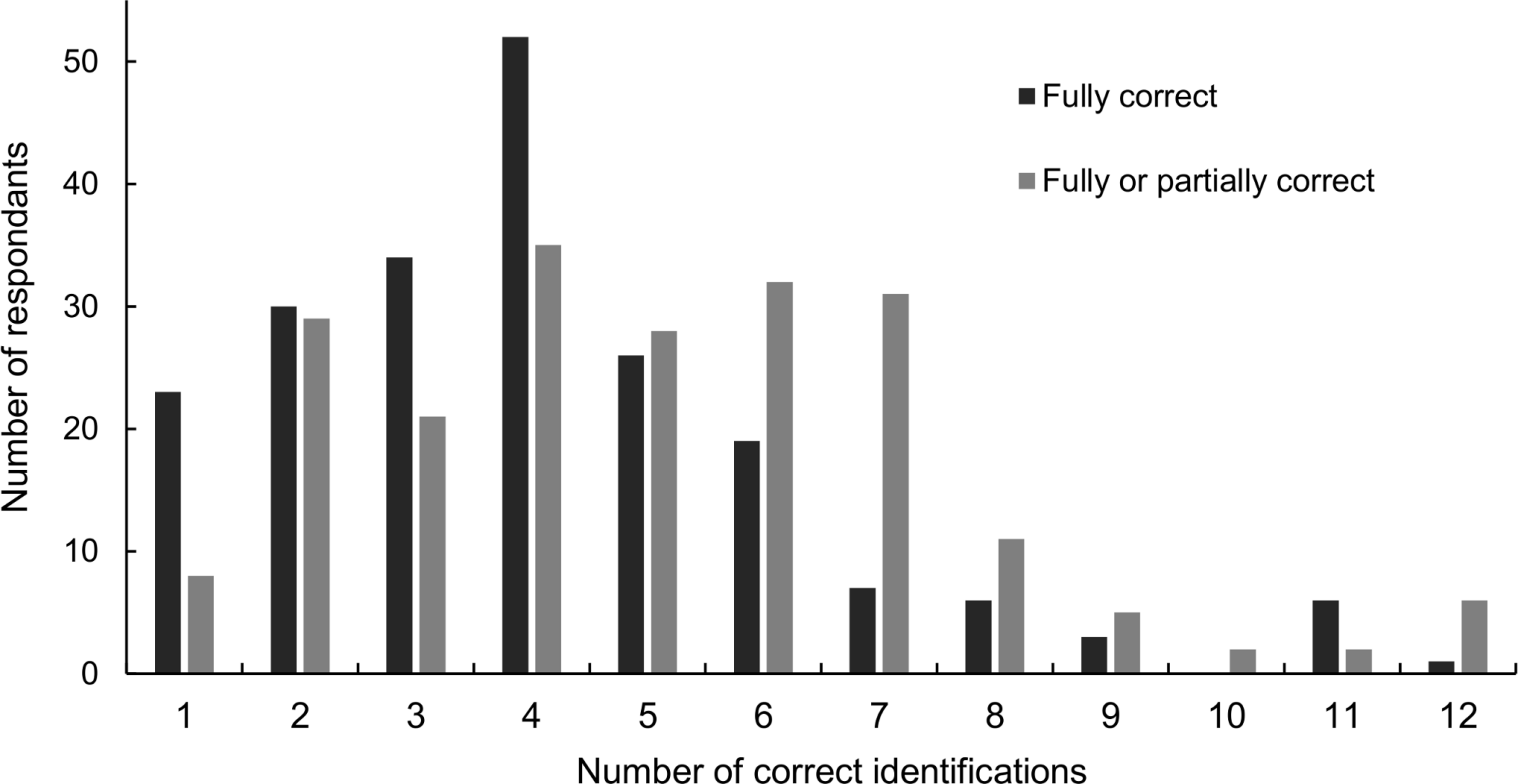
Number of fully and partially correct bird identifications (pre-project). Dark grey bars = fully correct; light grey bars = fully or partially correct. Not included here are the children who could not fully (n = 11) or partially (n = 8) identify a single species.

Before the project, 98 (45.0%) of children could correctly name three or fewer common bird species–decreasing to 66 (30.3%) children if including partially correct answers (Fig 1). Baseline bird identification ability was higher in children that were female, whose family fed birds in their garden all year round, owned a pet, and engaged in more nature-based activities (Table 1). However, this best model had a marginal R^2^ of 0.03 and a conditional R^2^ of 0.08.

**Table 1:** Summary of minimum adequate (top candidate) GLMM for pre-project bird identification ability.

	Estimate	Std. Error	Z value	P-value
Binomial error structure and logit link; n = 202; AIC = 1360				
(Intercept)	-1.084	0.174	-6.236	<0.001
Gender (male)	-0.289	0.065	-4.448	<0.001
Pets (yes)	0.134	0.068	1.978	0.048
Feed birds (sometimes)	0.050	0.077	0.653	0.513
Feed birds (all year)	0.314	0.094	3.331	<0.001
Feed birds (no garden)	0.110	0.123	0.892	0.372
Nature activities	0.094	0.014	6.852	<0.001

Base categories for the retained factors were: female; no pets; and if the participant’s family did not feed birds in their garden. School is a random factor.

**Fig 2:**
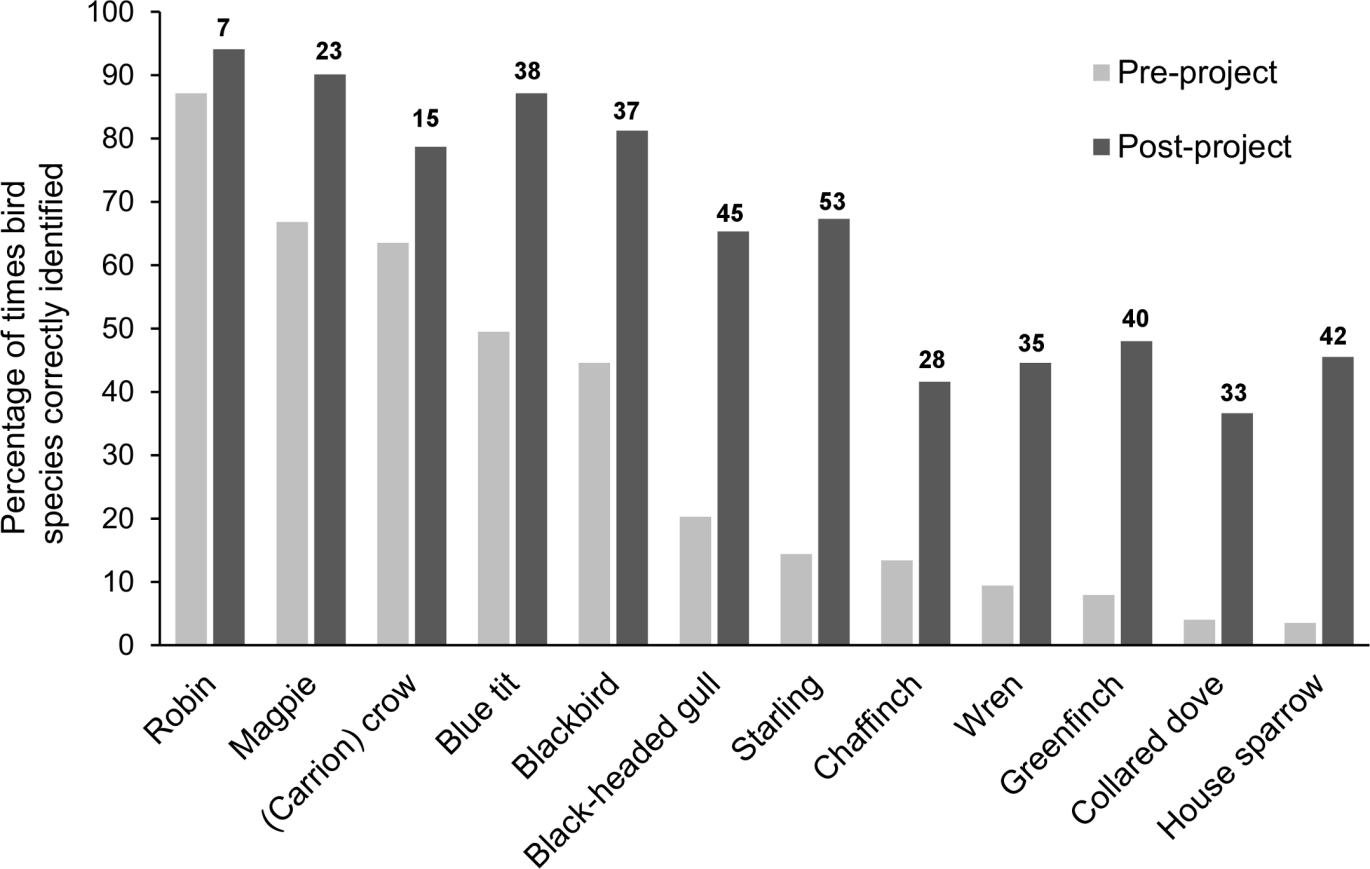
Percentage of times each bird species was correctly identified (i.e. scored 2 points). Light grey bars = pre-project; dark grey bars = post-project. Numbers above bars correspond to percentage change in score.

Bird identification ability increased significantly from an average score of 8.7 (SD ± 5.0) pre-project to 16.5 (SD ± 5.9) post-project (paired Wilcoxon signed-rank test: n = 202, w = 618.5, *P* = <0.001). This increase in identification score was also found within each sociodemographic factor (Table C in S5 Appendix). A total of 177 (87.6%) respondents scored higher post-project, 9 (4.5%) obtained no score change, and 16 (7.9%) scored lower. Proportion of correct identifications increased from pre- to post-project for each of the 12 bird species, but identification ability varied markedly by species (Fig 2). The top five bird species fully identified to common species level (pre- and post-project) were: robin, magpie, (carrion) crow, blue tit and blackbird. House sparrow and collared dove were the hardest birds to fully identify pre-project, and chaffinch and collared dove hardest post-project (see Table D in S5 Appendix for the proportion of correctly identified species for each school, year group and species).

Both the total number of misidentifications (pre = 450, post = 319) and number of blank answers (pre = 1189, post = 514) considerably decreased post-project. Most common pre-project mistakes were identifying the black-headed gull as a (sea) gull (n = 97), collared dove as a pigeon (n = 40) and blackbird as (carrion) crow (n = 24). Most common post-project mistakes were misidentifying the collared dove as a (wood) pigeon (n = 36), only identifying the house sparrow as a sparrow (n = 25) and calling the black-headed gull a herring gull (n = 24) or simply (sea) gull (n = 22). There were an equal number of “made-up” species pre- and post-project (16 and 14, respectively) and 12 non-native species given as answers pre-project (whereas no exotics were referred to post-project). A shift from unspecific (e.g. seagull) to more specific (e.g. black-headed gull) identifications was identified (see Tables E and F in S5 Appendix for full breakdown of misidentifications).

Post-project bird identification knowledge was higher in children from year 3, who had a garden, and scored high in the pre-project test (Table 2A), with a marginal R^2^ of 0.12 and a conditional R^2^ of 0.16. Change in score was higher in children from year 3 and who, prior to the project, did not feed birds in their garden or engage in as many nature activities (Table 2B), with a marginal R^2^ of 0.25 and a conditional R^2^ of 0.30.

**Table 2:** Summary of minimum adequate (top candidate) GLMMs for (a) post-project bird identification ability and (b) score change.

	Estimate	Std. Error	Z value	P-value
**a) Post-project bird knowledge (Binomial error structure and logit link; n = 202; AIC = 1639)**				
(Intercept)	0.558	0.298	1.877	0.061
School year (year four)	-1.048	0.379	-2.767	0.006
School year (year five)	-1.223	0.331	-3.696	<0.001
Outdoor space (yes)	0.297	0.123	2.406	0.016
Pre-project knowledge	0.107	0.008	12.557	<0.001
**b) Change in bird knowledge (Gaussian error structure and identity link; n = 202; AIC = 1263.2)**				
(Intercept)	15.171	1.477	10.271	<0.001
School year (year four)	-5.536	1.746	-3.170	0.002
School year (year five)	-7.074	1.554	-4.553	<0.001
Feed birds (sometimes)	-0.632	0.882	-0.716	0.474
Feed birds (all year)	-2.587	1.116	-2.319	0.020
Feed birds (no garden)	-1.495	1.397	-1.070	0.285
Nature activities	-0.356	0.156	-2.277	0.023

Base categories for the factors were: school year three, no outdoor space and if the participant’s family did not feed birds in their garden. School is a random factor in all models.

### Attitudes towards birds

When asked “how do you feel about birds?” 75.7% said they “(really) liked” birds pre-project compared with 82.2% post-project (paired Wilcoxon signed-rank test: w = 912, *P* = 0.002, n = 202). Overall, 51 children’s feelings towards birds became more positive, 126 experienced no change, and 25 a negative change. A significant positive relationship was found between feelings towards birds and knowledge both pre-project (POLR: t-value = 4.625, p<0.001) and post-project (POLR: t-value = 2.705, p<0.001) (n = 201). Since the project started, 123 (60%) of the students reported to have been bird-watching in some capacity outside of school, of which 39% had not been prior to this project—85.4% found the experience “(really) fun” (only two responded that they did not enjoy it).

Baseline composite (i.e. affect and utility) attitudinal scores towards birds were higher in those with higher pre-project identification scores and who engaged in more nature activities, with a marginal R^2^ of 0.05 and a conditional R^2^ of 0.16 (Table 3A). Composite attitude significantly increased post-project for all respondents combined (paired Wilcoxon signed-rank test: n = 167, w = 1443.5, *P* = <0.001), and this change in score was higher for females and children from Year 3, with both a marginal and conditional R^2^ of 0.11 (Table 3B).

**Table 3:** Summary of minimum adequate (top candidate) GLMMs for (a) pre-project composite attitude score and (b) score change.

	Estimate	Std. Error	Z value	P-value
**a) Pre-project composite attitude (Gaussian error structure and identity link; n = 167; AIC = 106)**				
(Intercept)	-0.615	0.070	-8.779	<0.001
Pre-project knowledge	0.009	0.005	1.697	0.050
Nature activities	0.019	0.010	1.804	0.041
**b) Change in composite attitude (Gaussian error structure and identity link; n = 167; AIC = 796)**				
(Intercept)	2.891	0.509	5.678	<0.001
School year (year four)	-1.118	0.572	-1.953	0.051
School year (year five)	-2.072	0.525	-3.944	<0.001
Gender (male)	-0.840	0.401	-2.095	0.036

Base categories for the factors were: school year three and female. School is a random factor in both models.

Composite attitudinal score significantly increased for all sociodemographic factor levels, except for four schools, Year 5 students, student’s with non-grass covered or no outdoor space, and those who had not seen birds in their outdoor space (Tables G and H in S5 Appendix). When analysed separately, both affect and utility scores also significantly increased post-project for all respondents combined (affect: n = 167, w = 103, *P* = 0.005; utility: n = 167, w = 1687, *P* = <0.001). By sociodemographic factor, significant increases in utility scores closely mirrored those for composite attitude scores, whereas fewer significant increases were found for affect scores (Tables G and H in S5 Appendix). Variation in composite, affect and utility scores across levels within each sociodemographic factor are summarised in Table I (S5 Appendix).

Pre- and post-project composite attitude score were significantly positively correlated (tau = 0.23, p = <0.001) as were pre- and post-project affect (tau = 0.35, p = <0.001) and utility (tau = 0.18, p = 0.007) scores. High affect scores related to high utility scores (pre-project: tau = 0.31, p = <0.001; post-project: tau = 0.25, p = <0.001). Additionally, significant positive correlations were found between identification score and composite attitude score (pre-project: tau = 0.19, p = 0.001; post-project: tau = 0.16, p = 0.011), but not for their change in scores (tau = 0.11, p = 0.059).

### Student project evaluation

**Fig 3:**
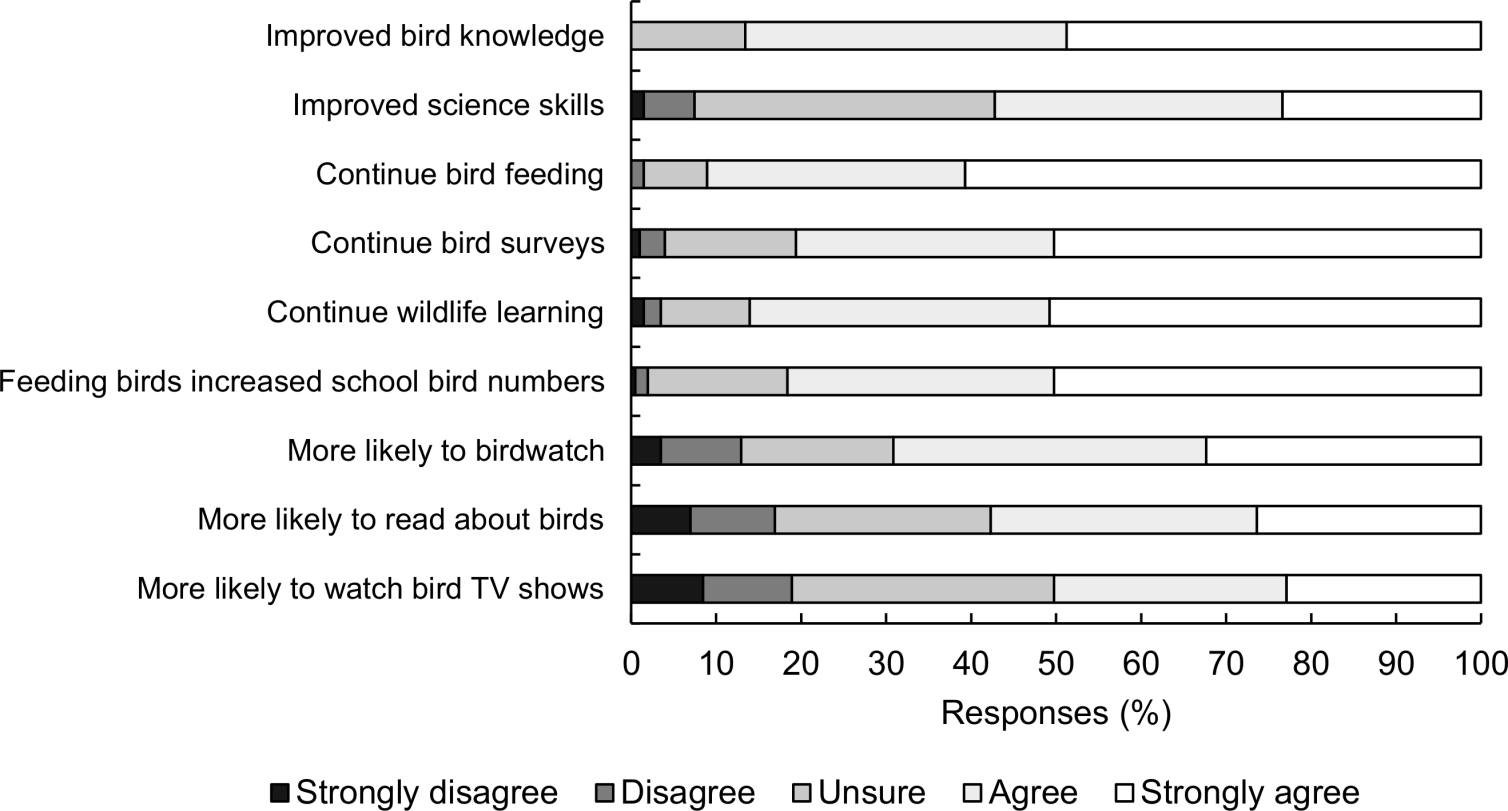
Responses by student participants to likert-style questions evaluating project outcomes. Only respondents who answered all nine items are included; n = 201.

A total of 201 students completed all nine items required to generate a student evaluation score for the project, with an average score of 36.4 (SD = 5.8, min. = 18, max. = 45) out of 45. Based on the proportion selecting “Agree” or “Strongly agree”, 87% and 57% believed the project had improved their bird knowledge and science skills, respectively. Within school, more than 90% wished to continue feeding birds, more than 80% wanted to carry-on surveying, and 86% wanted to continue learning about local wildlife. Slightly lower proportions felt that, as a result of this project, they are more likely to go birdwatching (69%), read about birds (58%) or watch TV shows on birds (50%). More than 80% believed that the number of birds in their school grounds had increased (see Fig 3 for overall responses and Table J in S5 Appendix for a breakdown by school).

When asked “what do you think of this bird project?” no students “(really) disliked” it and 86% deemed it “fun” (n = 61) or “really fun” (n = 111), with all schools scoring an average of 4.0 or above on the five-point Likert scale used. In describing what they liked most about the project, student’s answers could be split into eight themes: (a) bird watching/surveys (n = 79), (b) feeding birds (n = 39), (c) learning bird identification and recognising birds outside (n = 16), (d) surveys where you saw many birds (n = 10), seeing new species (n = 9), (e) listening to birds singing (n = 9), (f) seeing birds eat the bird food (n = 8), (g) being outside (n = 6), and (h) helping local wildlife (n = 2). A total of 38 students explicitly wrote that they enjoyed all elements of the project.

Examples of positive quotes from participating students include: “It was really good that you can see new birds that you might not see if you don’t look. It makes you relax and look and be calm”. Another wrote “I can remember when we first went out and I spotted a chaffinch… When we went out for the last time a house sparrow came really close. Every bird was amazing”. Whilst another noted “We didn’t see many birds with no food out, then when we put food in the feeders we saw lots of birds and seeing the birds was what I liked the most”.

Aspects of the project that students liked least could be categorised into seven themes: (a) not seeing any/more birds when surveying (n = 27), (b) having to wait (in cold)/keep quiet and still during surveys (n = 26), (c) pre-feeding surveys (n = 17), (d) noise from other classes/members of survey groups (n = 14), (e) identifying/counting birds because it was difficult (n = 10), (f) cleaning/refilling bird feeders (n = 8), and (g) not being able to do more birdwatching. However, 40 students explicitly stated disliking no aspect of the project.

### Teacher project evaluation

All teachers (n = 8) completed all items of the immediate project evaluation questionnaire. Average teacher evaluation score was 58.1 (*SD* = 4.2) out of 65, indicating positive endorsement of the project (Table 4). More responses scored “(strongly) agree” than “(strongly) disagree” for each statement, except when asked if the project was time-consuming. Here, answers ranged from “strongly agree” to “strongly disagree” and were split with 50% agreeing and 50% disagreeing.

**Table 4:** Immediate post-project evaluation scores from teachers (n = 8).

Question	Mean score (+/- SD)	Number (%) who (strongly) agree
Information for teachers were comprehensive	5.0 (0.0)	8 (100)
I felt prepared and confident running the project with my class	5.0 (0.0)	8 (100)
Engagement workshop was well delivered and enjoyed by class	4.8 (0.5)	8 (100)
Project resources provided were age-appropriate	5.0 (0.0)	8 (100)
Class learned about birds from this project	4.6 (0.5)	8 (100)
Class enhanced their science skills from this project	3.9 (0.6)	6 (75)
Class wants to continue bird feeding/watching	4.5 (0.8)	7 (88)
Class used the educational materials frequently during project	4.0 (0.8)	6 (75)
The project was time consuming^a^	2.9 (1.6)	4 (50)
I would recommend this workshop to other teachers	4.4 (0.7)	7 (88)
		**Number (%) who scored excellent or good**
Pupil enjoyment / interest level	4.5 (0.5)	8 (100)
Project organisation	4.8 (0.5)	8 (100)
Quality of resources and equipment provided	4.9 (0.4)	8 (100)

5 = Strongly agree / Excellent, 1 = Strongly disagree / Poor.

^a^ Statement was reverse scored.

Focusing on responses to the open-ended questions, motivations for involvement in the project can be categorised into four themes: (1) increase student’s awareness and knowledge of local (avian) biodiversity, (2) increase student’s awareness of environmental concerns and importance of environmental stewardship, (3) increase utilisation of school grounds by both students and wildlife, and (4) to make the subject of (applied) science a higher priority/complements and links with existing curriculum.

In describing what they liked most about the project, all teachers commented on their student’s enthusiasm and excitement in conducting the surveys and learning in an outside environment. Most also mentioned noticing an observed increase in student’s awareness, knowledge and interest in wildlife. For example, one teacher wrote: “Before the project, the enthusiasm was mixed throughout the class but during the project, every single child in the class has been fully engaged. Children come into school on a Monday morning discussing which birds they saw over the weekend and which bird calls they may have heard” (see S6 Appendix for additional open-answers from teachers). Regarding aspects of the project participating teacher’s liked least, a couple highlighted that it was either time consuming, an additional workload for them, or sometimes difficult to schedule around taught activities. One commented that during the supervised bird surveys their assistant could not help other children in class. One mentioned difficulties with squirrels at the feeders, while two commented on the lack of bird activity/different species observed during surveys. Two teachers explicitly stated that there were no aspects of the project that were not enjoyed by them or the class.

Anticipated long-term benefits from participating in the project were varied, including: re-utilising resources to feed and monitor birds, promotion of active/outside learning that complements more traditional teaching, and instilling in students a greater appreciation and understanding of their surroundings and what wildlife lives in it. For example, one teacher commented that “For some, it is likely to have instilled a lifelong interest in birdwatching” (see S6 Appendix for additional open answers from teachers). All participating teachers were keen to run a similar project in the future.

### Long-term follow-up

Based on responses from six (75%) of the participating teachers, a year after the project, all but two reported that the class/school were still (regularly) feeding birds in their school grounds, with one teacher commenting that additional feeders had been made. Time constraints and not enough teaching assistants were provided as reasons for the two schools no longer feeding birds, with one adding “pressures on the curriculum are too high”. All schools still feeding birds also continued birdwatching, with one school continuing surveys and two schools still using the educational materials. One class shared their findings with other classes so that they can also birdwatch. As a direct result of “Bird Buddies”, two schools reported actively participating in the RSPB’s Bird Schools’ Birdwatch, with another school making habitat for bees and planting wildflowers. One of the teachers stated that “Those in the original class are generally more interested and involved in any school-based environmental activity–it has made them think and look around them more, and it’s fantastic they’re still proactively feeding birds”.

## Discussion

Providing environmental education opportunities to urban children is a potential tool to mediate the current extinction of experience with the natural world. There are multiple benefits of outdoor learning within school grounds, including the close vicinity and easy access to school (saving money on travel costs and valuable teaching time), ease of frequent visits (facilitating long-term studies), and the realisation that children are more attentive to educational tasks, learn more, and feel more comfortable when taught in a familiar rather than novel environment [58,60]. Furthermore, exploring biodiversity in school surroundings can target children traditionally unreached by science outreach or biodiversity-related volunteering programs [9]. To the best of our knowledge, this is the first study to explicitly examine children’s awareness, knowledge and attitudes towards local wildlife before and after active participation in a school-based supplementary feeding and monitoring project (“Bird Buddies”).

This study found increased awareness and interest in local wildlife amongst participating children, which is a reassuring outcome given numerous research indicating that many urban-living people are unaware of the nature around them [81–82]. More than 80% of children believed the number of birds had increased within their school grounds as a direct result of this project, despite inconclusive support from the bird survey data. This supports findings from existing studies suggesting people are not consciously aware of most components of biodiversity [81,83], and that environmental education initiatives can provide such “eye-opening” opportunities [84].

Recognition of common bird species prior to the project was poor, despite birds being comparatively conspicuous and charismatic, reaffirming concerns about taxonomic illiteracy and loss of environmental knowledge (e.g. [9,29–32]). This inability, at least in part, reflects that little time nowadays is spent in school on direct observation of nature [67,70]. Acknowledging inconsistent evidence to date regarding gender differences in ecological learning, our study found females to have higher baseline species identification ability than males, and, in agreement with previous studies, we found pet owners [34,37] and those who engaged in more nature-based activities [38] to be more knowledgeable. Supporting the findings of Beck et al. [53], we also found children from families that feed birds at home had greater baseline knowledge, potentially due to increased exposure to wild birds and/or knowledge transfer from family members.

Participation in the project increased the ability to distinguish between local bird species, with 88% of children improving their knowledge scores. This was as a combined result of the project engagement workshop, interactive activities and reference resources, and repeat engagement in both bird feeding and monitoring. First-hand outdoor experiences are purported to enhance memory and knowledge retention [85]. Improved knowledge was also found in comparable initiatives (e.g. [53,68,86], and, as stated by Balmford et al. [29]: “children clearly have tremendous capacity for learning about wildlife”. Pre-project knowledge score was the strongest predictor of corresponding post-project knowledge, with both younger children (i.e. Year 3: 7–8 year olds) and having a garden at home also significant final model factors; the latter potentially facilitated birdwatching and subsequently learning from home. The youngest children were also found to obtain the biggest change in identification score (as in 54,68). Despite uncertainties as to why (although see [33]), these results suggest educational efforts aiming to improve knowledge of animals should target younger primary school children. Additionally, significant positive change in identification score was obtained by children who, prior to the project, had little contact with nature (i.e. did not feed birds or participate in as many nature activities). This relates to a recent study by Cox & Gaston [71] who found a strong correlation between the number of bird species adults could correctly identify and how connected to nature they felt.

A number of studies have emphasised that children’s lack of knowledge on local species compared with exotics may be due to over-representation of the latter across various media (e.g. [9,87–88]). We find that although misidentification of species to exotics was relatively common pre-project, no non-native species were listed at all post-project. Further detail on the nature of improved knowledge found in our study includes children listing general taxonomic units (i.e. pigeon, gull) less often than specific species (i.e. collared dove, black-headed gull) post-project, as in previous studies [40]. Misconceptions about species, including birds, are common amongst school children [89] and, if not addressed, can persist into adulthood [90].

It is important to highlight that, although correct identification increased for all bird species, identification ability varied across species (see also [71]). It is unsurprising that the robin was the most recognisable species given its popularity and significance within UK culture. Ability to correctly identify species in this study is likely related to multiple factors, including: colour, ecology, behaviour, how locally common they are, and frequency of sightings during bird feeder surveys. For example, the house sparrow had very low baseline identification scores, which likely relates to their small size, brown colouration, lack of distinguishing features (especially females), and restless behaviour. However, after being told what they look like and all schools observing them at their bird feeders, identification scores increased considerably post-project. Interestingly, both the number and species of bird recorded by each class did not seem to influence overall or individual species post-project identification scores, suggesting this project facilitated learning via both direct (e.g. bird feeding, watching and surveying) and vicarious (e.g. bird games, class sightings poster, and talking to class members) experiences (see [6]).

Overall, children’s attitudes towards birds improved over the course of the project, with significant increases in likeability, affect, utility and composite attitude scores. Supporting our findings, but focusing instead on adults, Cox & Gaston [71] found likeability of birds to be higher for people who feed birds regularly. Similarly, baseline affect scores were very high (compared to utility), suggesting children aged between 7 and 10 are better at forging a positive affective and/or emotional response to birds than appreciating their instrumental value [33]. For all metrics of attitude used in this study, we pooled bird species together; however, it is important to highlight that previous studies have found attitudes to vary towards different bird species and groups (e.g. [52,71,91]). Baseline composite attitude was highest in children who participated in more nature activities (in agreement with Soga et al. [6]) and had higher baseline identification scores (see later discussion). Although only significant in univariate analyses, it is interesting to highlight that baseline attitude was also higher in those who saw birds in their outdoor space and whose family fed birds prior to the project, suggesting that experience with nature facilitates positive environmental attitudes. Change in attitude was higher in younger children and females, the former trend supporting the theory that younger children are more responsive to environmental education, concerning positive attitude shifts, than older children [92]. In addition, a ceiling effect may be partly responsible for attitudes not improving for all sociodemographic groups/levels and for a lack of variation in post-project attitude scores (which were severely negatively skewed). For example, Beck et al. [53] partly attributed a lack of attitudinal change in their home bird-feeding project to the already relatively high environmental attitudes of their child participants.

Random effects in all models were significant, suggesting students who are in the same class have knowledge and attitudes that are more similar than can be attributed to random chance. This realisation provides support for the use of multivariate approaches in such studies, which have rarely been used to date. Furthermore, a considerable amount of variation remained unexplained in our models investigating knowledge and attitudes (particularly pre-project knowledge), suggesting additional unexplored contributing variables; e.g. school grades, family, and media [9].

Although determining drivers of attitudinal change is inherently complex, knowledge can facilitate attitude formation [23–24]–“people care about what they know” [29]. We found bird likeability and knowledge to be positively related (see also [71]), and, despite change in bird knowledge being uncorrelated with attitudinal change towards birds, baseline knowledge was a key positive predictor of baseline attitudes and post-project knowledge and attitudes were positively correlated. This positive relationship between environmental knowledge and attitude has been reported previously (e.g. [34,48,93–94]), and although a causal relationship cannot be confirmed here, it does suggest that the two are at least linked. Overall, we found knowledge increased more than attitudes (see also [25]) and, although this may partly be due to a ceiling effect with attitude, the relative ability for environmental education to foster both knowledge and attitude gains warrants further research. An important point to consider with environmental education projects is that acquired knowledge may not last long, with projects solely based on knowledge acquisition often inefficient in driving attitude change. Although the design of our study could only establish short-term impacts, projects like “Bird Buddies” which have a mixture of activities promoting personal responsibility on the success of their outcomes (e.g. set-up and maintenance of bird feeding stations and monitoring of birds visiting them) have the potential to maintain acquired knowledge for longer and thus effectively contribute to attitude development and change [95].

A key outcome from this project was that both knowledge and attitudes improved most for children with the least prior exposure to nature, emphasising the important role that schools can play in reconnecting children with nature. In fact, as our respondent sample was biased towards children with outdoor space at home and at least some prior experience with nature, it can be predicted that greater changes in knowledge and attitude are likely to be found for schools engaging in the project in the future where children have even less baseline exposure to nature.

Pro-environmental behaviour is an implicit desired outcome from environmental education projects. Although not explicitly studied here, we found some indication of actual behavioural change after participation in the relatively short duration (six weeks) “Bird Buddies” project. For example, 39% of children who went birdwatching outside of school since the project started had not been before. We also found evidence of intentions or desires to act in the future, with most children declaring they want to continue feeding and surveying birds at their school and to engage in both direct and vicarious bird experiences at home. The long-term follow-up, one year after the project, also provided positive evidence that some schools were still engaging in project-related activities, with a couple also involved in other environmental education initiatives and further school-ground improvement as a direct result of “Bird Buddies”. We also found some anecdotal evidence of knock-on (i.e. “ripple”) effects, with children and teachers sharing their knowledge and attitudes with friends, family members and the rest of the school community (e.g. by running special school assemblies, training other classes, and asking parents to put bird feeders up at home; see S6 Appendix).

Feeding birds in gardens is a very popular past time for adults, and this project increased children’s attitudes towards birdwatching. Although not explicitly studied here, in addition to reconnecting people to the natural world, there is growing evidence that watching, listening to and feeding birds can have positive health and wellbeing benefits [81,96–98]. Bird feeders, in particular, act as focal points for delivery of these benefits [71,96], facilitating the ability to regularly see and distinguish between different species at close proximity, and so appreciate local wildlife and diversity. For example, several children explicitly stated that the project made them feel calm and relaxed (see also [99]). Investigating the health and wellbeing benefits that children derive from outdoor nature activities such as birdwatching is an important future research area.

The successful improvement of environmental education programmes requires both short- and long-term evaluation of their outcomes, alongside collating participants’ feedback, and identifying unanticipated events or barriers. Nevertheless, there are limited such assessments to date. As discussed by Randler & Bogner [40], despite their recognised value, outdoor educational settings for birds are often neglected by schools due to various perceived difficulties. However, “Bird Buddies” obtained positive evaluations from both children and teachers, showing that such an education approach is both feasible and appreciated–joining a small but growing evidence-base highlighting the positive effects of outdoor direct experiential learning (e.g. [53,57,70]). Children reported enjoyment throughout the project and, although a positive outcome, it is important to emphasise that learning and enjoyment are not always compatible [58]. It is therefore vital that environmental education projects result in both learning gains and a positive educational experience for students–as shown here. In the present study we were able to conduct a long-term follow-up with the participating teachers, but not the individual students themselves due to logistical constraints (e.g. students changing class/year or moving school). As discussed in Shwartz et al. [56], where possible, long-term follow-ups should try to conduct repeat surveys of individual participants in order to establish to what extent obtained outcomes are retained.

Although teachers positively evaluated the project, several constraints were reported, mainly relating to shortages of time, difficulties in scheduling around other teaching commitments, and lack of teaching assistant support. These partly overlap with general barriers to the provision of outdoor learning by schools collated by Rickinson et al. [60]. Full cooperation and enthusiasm from teachers is essential, and we facilitated this by providing all required resources, training, clear written instructions and keeping daily bird surveys to 10 minutes. Nevertheless, reported constraints occasionally meant bird surveys were conducted outside optimal conditions (S1 Appendix) or that staff (rather than children) filled/cleaned the bird feeders or entered survey data online. Although such deviations from the project protocol have the potential to influence outcomes, and should be reported to promote transparency, due to the low frequency of their occurrence the impact was likely negligible. Teacher satisfaction is very important for projects such as “Bird Buddies”, which are designed as an educational supplement and voluntarily included in teaching programmes, as the willingness of teachers to continue such projects depends on their feeling that it was a success [70]. Positively, all teachers expressed a desire to run a similar project in the future. It is important to note that the participating teachers may not be representative of teachers in general, as it is highly probable they are particularly interested in environmental education, having volunteered to incorporate the project into their timetable. Similarly, more research is needed to increase our understanding of the effects of such environmental education projects in different geographical and cultural contexts.

In an attempt to reconnect children with nature, school curricula are being updated to include requirements for students to have knowledge and understanding of the natural world (e.g. [100–101]). Biodiversity fits well within science and geography curricula, but can be easily integrated into other subjects. For instance, the survey data generated by “Bird Buddies” can be used to support numeracy and computing lessons, and a couple of participating teachers reported using the project’s focus within art and creative writing lessons (see also [86]). Outputs from such monitoring projects can also contribute to wider citizen science projects; e.g. on supplemental feeding of birds (see [98]). Many urban schools are “nature deprived”; however, simple steps can enhance their biodiversity value. Converting school grounds into ecologically valuable landscapes would enable them to be used as a multipurpose resource for study, recreation and aesthetics [102], whilst acting as part of urban wildlife corridors.

## Conclusion

The increasing disconnect between people and nature is greatest in urban areas and among children. This study showed that urban environmental education within school grounds can increase children’s awareness, knowledge and attitudes towards local biodiversity–specifically, projects that promote action-based experiential learning via direct wildlife experiences. Importantly, “Bird Buddies” was found to engage children who previously had little experience with nature, emphasising the important role that schools can play in reconnecting children with nature. Collectively, the findings from this study, including positive student and teacher evaluations, support schools in urban areas making wider use of such locally-based environmental education projects–not only for educational gains as shown here, but also for potential health, wellbeing and biodiversity benefits. Ultimately, the outputs from this study provide a hopeful message that, even in a rapidly urbanising world, a simple, relatively inexpensive and non-time-consuming initiative can help towards reducing the extinction of experience and reconnecting children with nature.

## Supporting information

S1 Appendix “Bird Buddies” engagement workshop and bird monitoring protocols. (DOCX)

S2 Appendix Pre- and post-project questionnaires and associated coding. (DOCX)

S3 Appendix Teacher project evaluation and long-term follow-up questionnaires. (DOCX)

S4 Appendix Complete closed-answer pre- and post-project questionnaire results. (XLSX)

S5 Appendix Supplementary results. Summary statistics for sociodemographic attributes of participants **(Table A)**. Species included in identification test and seen during bird surveys (**Table B).** Results of paired Wilcoxon signed rank tests between pre- and post-project bird identification scores per sociodemographic factor **(Table C)**. Percentage of children at each school and year group that correctly identified bird species pre-project and post-project **(Table D)**. Species misidentifications in pre- and post-project tests **(Table E)**. Number of species misidentifications and blank answers in pre- and post-project tests per species **(Table F)**. Results of paired Wilcoxon signed rank tests between pre- and post-project composite, affect and utility attitude scores per sociodemographic factor **(Table G)**. Descriptive statistics for pre- and post-project attitude scores per sociodemographic factor (**Table H**). Results of Mann-Whitney or Kruskal-Wallis tests per sociodemographic factor for pre- and post-project attitude scores and change in score **(Table I)**. Proportion of children per school that scored either “Agree” or “Strongly agree” for student evaluation statements (**Table J**).
(DOCX)

S6 Appendix Example open-answers from teacher project evaluation questionnaire. (DOCX)
