# Supplementary material for: Birds in the playground: Evaluating the effectiveness of an urban environmental education project in enhancing school children’s awareness, knowledge and attitudes towards local wildlife
Source: PLoS One. 2018 Mar 6;13(3):e0193993. doi: 10.1371/journal.pone.0193993 (PMC5839573; doi:10.1371/journal.pone.0193993)
Supplement: S1 Appendix — (DOCX) [file pone.0193993.s001.docx]

**S1 Appendix:** “Bird Buddies” engagement workshop and bird monitoring protocols

**Engagement workshop**

A “Bird Buddies” project introduction and engagement workshop was run with each participating class lasting, on average, 90 minutes. This comprised of the following three elements:

1. A short (20 minute) interactive and visual presentation introducing the project to the school children, the importance of bird feeding, and how to identify different common UK bird species (via photos and bird-song).
2. Group-based educational and engagement activities centred on bird identification and ecology. The class was split into three groups with each playing one of bird bingo, matching pairs of bird illustrations, or matching bird illustrations with photos and species names. All birds used in these activities were common UK species, including those tested in the questionnaires. Each group were asked to teach their fellow class members their activity at a later date.
3. A short (five minute) practice bird survey using video footage of birds at a bird feeder (see section below for protocol followed).

Each class kept the interactive activities used during the workshop, and were encouraged to use them during breaks and “golden time” (or equivalent) throughout the project duration. Prior to the engagement workshop, the teachers from each participating class were provided with a “*Project Overview and Guidelines for Teachers*” booklet, concisely detailing all of the key information needed to successfully undertake the project.

**Bird monitoring protocol**

Each class was provided with the following bird feeding equipment and resources: two bird feeding stations (with three brackets for hanging feeders, a water dish and food dish), six assorted bird feeders, a selection of suitable bird food, bird food scoop and storage bin. Each class also received the following resources intended to assist with the bird surveys: a copy of the laminated FSC “*Guide to the top 50 garden birds*” fold-out chart and a “*School Bird Flashcard Booklet*”, the latter highlighting the most likely species to be seen during the surveys and comprising photos and several facts per species.

Instructions on how to set-up and maintain the bird feeders were provided in the teacher booklet. Two key pieces of advice were positioning the feeders so that they could be seen clearly (either from a window or sheltered spot outside), and in a safe location (for both birds and school children). Specifically, they were advised to position the bird feeders in a quiet area of the school grounds to enable birds to feed relatively undisturbed and, if possible, 3-4 meters away from natural shelter (e.g. shrubs and trees). Each class was instructed to monitor the food supply carefully (ensuring it was kept topped up), clean and wash the bird feeding station and feeders at least fortnightly, and to clean and refill water containers daily (but not immediately before a bird survey, to limit disturbance).

Bird surveys of 10-minutes duration were conducted 2-3 times per week by the participating school children in small groups of 5-6 (using a rota system). The groups were always supervised by a trained teaching assistant. Teachers were instructed that the surveys should be conducted in the morning, under fair weather conditions, and to avoid times when the school grounds could be noisy; i.e. during playtime and physical education sessions. Survey data were recorded on pre-designed data sheets and comprised of: week number, date, weather, start time, species observed and number seen. Only those birds that landed on or within the vicinity of the bird feeding stations were recorded. To ease data entry, the datasheets contained colour illustrations of 20 common UK birds (in the same style as those of the questionnaires). Participants simply had to circle the number under each species they saw that corresponded to the maximum number of individuals seen.

Bird surveys spanned a total of six weeks – two weeks before enrichment of the school grounds (i.e. set-up of the bird feeding station area) and four weeks during the enrichment. At the end of each survey week, data were transferred online using SurveyMonkey as a platform. Teachers were encouraged to let the school children assist/lead with this data entry. Teachers kept a record of which children took part in each survey, with the understanding that each should participate in at least one pre-feeding and two feeding surveys. A wall chart was provided per class for entering each survey’s data so that the whole class could see what was being sighted. If a student missed their assigned survey they would typically join the next scheduled survey. The project has been designed such that children not conducting a survey during a given week would still be engaged, due to the combination of direct (e.g. bird feeding, watching and surveying) and vicarious (e.g. bird games, sightings poster, and talking to class members) experiences.
