# Supplementary material for: Birds in the playground: Evaluating the effectiveness of an urban environmental education project in enhancing school children’s awareness, knowledge and attitudes towards local wildlife
Source: PLoS One. 2018 Mar 6;13(3):e0193993. doi: 10.1371/journal.pone.0193993 (PMC5839573; doi:10.1371/journal.pone.0193993)
Supplement: S2 Appendix — (DOCX) [file pone.0193993.s002.docx]

**S2 Appendix:** Pre- and post-project questionnaires and associated coding

**FRONT SHEET OF QUESTIONNAIRES**

**Instructions**

- Please answer ALL questions
- Answer the questions on your own
- Please read questions carefully
- If you are not sure what something means please ask your teacher

**Pre-entered information**

- School name
- Anonymous ID number (supplied by teacher)

**----------------------------------------------------------------------------------------------------**

**PRE-PROJECT QUESTIONNAIRE ITEMS (numbers refer to associated code for data analysis)**

**Q1:** Are you a boy or girl? *[Tick appropriate box]*

- Boy = 1
- Girl = 2

**Q2:** At home do you have outside space? *[Tick appropriate box]*

- Outside space with grass = 2
- Outside space without grass = 1
- No outside space = 0

*[also explored as a two-level factor: outside space = 1; no outside space = 0]*

**Q3:** Have you seen birds in your outside space? *[Tick appropriate box]*

- Yes = 1
- No = 0
- No outside space = 2

**Q4:** Does your family feed birds in your outside space? *[Tick appropriate box]*

- Yes – all year round = 2
- Sometimes = 1
- No = 0
- My home has no outside space = 3

*[also explored as a three-level factor: yes = 1; no = 0; no outside space = 3].*

***Q5:** How do you feel about birds? *[Tick appropriate box]*

- Scored on a five-point Likert scale: 5 = “I really like birds”, 4 = “I like birds”, 3 = “Birds are OK”, 2 = “I don’t like birds”, 1 = “I really don’t like birds”.

**Q6a:** Do you have any pets? *[Tick appropriate box]*

- Yes = 1
- No = 0

**Q6b:** If yes, what are they and how many? *[Open answer question]*

- Dog = 1, cat = 2, hamster = 3, rabbit = 4, fish = 5, gerbil = 6, reptile = 7, bird = 8, guinea pig = 9, horse = 10, chicken = 11

**Q7:** Which of the following activities have you done in the past year? *[Tick appropriate boxes - score out of 10]*

| 1. Watching birds | 1. Nature walking |
| --- | --- |
| 1. Wildlife/environment/outdoors club | 1. Horse riding |
| 1. Read books/online about wildlife | 1. Fishing |
| 1. Watch wildlife TV shows | 1. Watching wildlife |
| 1. Visit parks/countryside/beach | 1. Visit zoo/aquarium |

**Q8:** If you have ever been bird-watching, how did you feel about it? *[Tick appropriate box]*

- Scored on a five-point Likert scale: 5 = “It was really fun”, 4 = “It was fun”, 3 = “It was OK”, 2 = “I didn’t like it”, 1 = “I really didn’t like it”.

***Q9:** For each of the questions in the box below, please tick either yes or no *[Text in italics not provided to participants]*.

| ***Question*** | ***Axis*** | ***Score if answered ‘yes’*** |
| --- | --- | --- |
| 1. Do you like birds? | *Affect* | *1* |
| 1. Are you scared of birds? | *Affect* | *–1* |
| 1. Can people make money from bird watching? | *Utility* | *1* |
| 1. Are wild birds a pest? | *Utility* | *–1* |
| 1. Would you be sad if birds no longer existed? | *Affect* | *1* |
| 1. Are birds important to keep the environment healthy? | *Utility* | *1* |
| 1. Do you hate birds? | *Affect* | *–1* |
| 1. Would people be better off if birds no longer existed? | *Utility* | *–1* |

***Q10:** Write the name of the birds below each picture. *[All pictures comprised of a colour illustration of an adult individual. For species that display sexual dichromatism, only the male was shown – score out of 24].*

1. **Blue tit** [“Blue tit” = 2 points; “Tit” = 1 point; any other tit species = 1 point]
2. **Robin** [“Robin” = 2 points; nothing else accepted]
3. **House sparrow** [“House sparrow” = 2 points; “Sparrow” = 1 point; any other sparrow = 1 point]
4. **Chaffinch** [“Chaffinch” = 2 points; “finch” = 1 point; any other finch species = 1 point]
5. **Greenfinch** [“Greenfinch” = 2 points; “finch” = 1 point; any other finch species = 1 point]
6. **Wren** [Wren = 2 points; nothing else accepted]
7. **Blackbird** [Blackbird = 2 points; nothing else accepted]
8. **Starling** [Starling = 2 points; nothing else accepted]
9. **Magpie** [Magpie = 2 points; nothing else accepted]
10. **Collared dove** [Collared dove = 2 points; any other pigeon or dove species = 1 point]
11. **(Carrion) crow** [(Carrion) crow (2 pts). Any other black corvid (e.g. jackdaw, rook or raven) = 1 mark]
12. **Black-headed gull** [Black-headed gull = 2 points; (sea)gull and any other gull species = 1 mark]

** Questions asked in both the pre- and post-project questionnaires.*

**----------------------------------------------------------------------------------------------------**

**POST-PROJECT QUESTIONNAIRE ITEMS (numbers refer to associated code for data analysis)**

***Q1:** How do you feel about birds? *[Tick appropriate box]*

- Scored on a five-point Likert scale: 5 = “I really like birds”, 4 = “I like birds”, 3 = “Birds are OK”, 2 = “I don’t like birds”, 1 = “I really don’t like birds”.

**Q2a:** Have you been bird-watching outside of school since you have started this project? *[Tick appropriate box]*

- Yes = 1
- No = 0

**Q2b:** If yes, how did you feel about it? *[Tick appropriate box]*

- Scored on a five-point Likert scale: 5 = “It was really fun”, 4 = “It was fun”, 3 = “It was OK”, 2 = “I didn’t like it”, 1 = “I really didn’t like it”.

***Q3:** For each of the questions in the box below, please tick either yes or no.

| ***Question*** | ***Axis*** | ***Score if answered ‘yes’*** |
| --- | --- | --- |
| 1. Do you like birds? | *Affect* | *1* |
| 1. Are you scared of birds? | *Affect* | *–1* |
| 1. Can people make money from bird watching? | *Utility* | *1* |
| 1. Are wild birds a pest? | *Utility* | *–1* |
| 1. Would you be sad if birds no longer existed? | *Affect* | *1* |
| 1. Are birds important to keep the environment healthy? | *Utility* | *1* |
| 1. Do you hate birds? | *Affect* | *–1* |
| 1. Would people be better off if birds no longer existed? | *Utility* | *–1* |

***Q4:** Write the name of the birds below each picture. *[All pictures comprised of a colour illustration of an adult individual. For species that display sexual dichromatism, the male was shown – score out of 24].*

1. **Blue tit** [“Blue tit” = 2 points; “Tit” = 1 point; any other tit species = 1 point]
2. **Robin** [“Robin” = 2 points; nothing else accepted]
3. **House sparrow** [“House sparrow” = 2 points; “Sparrow” = 1 point; any other sparrow = 1 point]
4. **Chaffinch** [“Chaffinch” = 2 points; “finch” = 1 point; any other finch species = 1 point]
5. **Greenfinch** [“Greenfinch” = 2 points; “finch” = 1 point; any other finch species = 1 point]
6. **Wren** [Wren = 2 points; nothing else accepted]
7. **Blackbird** [Blackbird = 2 points; nothing else accepted]
8. **Starling** [Starling = 2 points; nothing else accepted]
9. **Magpie** [Magpie = 2 points; nothing else accepted]
10. **Collared dove** [Collared dove = 2 points; any other pigeon or dove species = 1 point]
11. **(Carrion) crow** [(Carrion) crow (2 pts). Any other black corvid (e.g. jackdaw, rook or raven) = 1 mark]
12. **Black-headed gull** [Black-headed gull = 2 points; (sea)gull & any other gull species = 1 mark]

**Q5:** For each of the statements below, please tick the box which you agree with. *[Scored on a five-point Likert scale: 5 = “Strongly agree”, 4 = “Agree”, 3 = “Unsure”, 2 = “Disagree”, 1 = “Strongly disagree”.]*

1. This project has improved my bird knowledge
2. This project has improved my science skills
3. I want to continue feeding birds at school
4. I want to continue surveying birds at school
5. I want to continue learning about local wildlife
6. I think feeding birds has helped increase the number of birds in our school grounds
7. Because of this project I am more likely to go bird-watching
8. Because of this project I am more likely to read about birds
9. Because of this project I am more likely to watch TV shows on birds

**Q6:** What do you think of this bird feeding project? *[Tick appropriate box]*

- Scored on a five-point Likert scale: 5 = “It was really fun”, 4 = “It was fun”, 3 = “It was OK”, 2 = “I didn’t like it”, 1 = “I really didn’t like it”.

**Q7:** Which part of the project did you like the least? *[Open answer question]*

**Q8:** Which part of the project did you like the most? *[Open answer question]*

** Questions asked in both the pre- and post-project questionnaires.*

**----------------------------------------------------------------------------------------------------**
