# Supplementary material for: Birds in the playground: Evaluating the effectiveness of an urban environmental education project in enhancing school children’s awareness, knowledge and attitudes towards local wildlife
Source: PLoS One. 2018 Mar 6;13(3):e0193993. doi: 10.1371/journal.pone.0193993 (PMC5839573; doi:10.1371/journal.pone.0193993)
Supplement: S3 Appendix — (DOCX) [file pone.0193993.s003.docx]

**S3 Appendix:** Teacher project evaluation and long-term follow-up questionnaires

**TEACHER PROJECT EVALUATION QUESTIONNAIRE**

**Q1:** What was your (school’s) motivation for getting involved in this project? *Type answer below*

**Q2:** For each of the statements below please highlight the response that best characterises how you feel about it with regards to this project. *[Please highlight your answer using the* ***Bold*** *or highlight function]*

|  | Strongly Agree | Agree | Neither Agree Nor Disagree | Disagree | Strongly Disagree |
| --- | --- | --- | --- | --- | --- |
| 1. Information for teachers (e.g. project instruction booklet and weekly emails) were comprehensive | 5 | 4 | 3 | 2 | 1 |
| 1. I felt prepared and confident running the project with my class | 5 | 4 | 3 | 2 | 1 |
| 1. Engagement workshop was well delivered and enjoyed by class | 5 | 4 | 3 | 2 | 1 |
| 1. Project resources provided were age-appropriate | 5 | 4 | 3 | 2 | 1 |
| 1. Class learned about birds from this project | 5 | 4 | 3 | 2 | 1 |
| 1. Class enhanced their science skills from this project | 5 | 4 | 3 | 2 | 1 |
| 1. Class wants to continue bird feeding/watching | 5 | 4 | 3 | 2 | 1 |
| 1. Class used the educational materials frequently during project | 5 | 4 | 3 | 2 | 1 |
| 1. The project was time consuming | 5 | 4 | 3 | 2 | 1 |
| 1. I would recommend this workshop to other teachers | 5 | 4 | 3 | 2 | 1 |

**Q3:** For each of the statements below please highlight the response that best characterises how you feel about it with regards to this project. *[Please highlight your answer using the* ***Bold*** *or highlight function]*

|  | Excellent | Good | Average | Disappointing | Poor |
| --- | --- | --- | --- | --- | --- |
| 1. Pupil enjoyment/interest level | 5 | 4 | 3 | 2 | 1 |
| 1. Project organisation | 5 | 4 | 3 | 2 | 1 |
| 1. Quality of resources and equipment provided | 5 | 4 | 3 | 2 | 1 |

**Q4:** Please comment on pupil’s enjoyment of project and what they have gained from it*. Type answer below*

**Q5:** Please add any anecdotal evidence/quotes from children to illustrate their engagement in the project. If parents have commented on their children’s involvement, please tell us how here. *Type answer below.*

**Q6:** What do you see as the long term benefits of the project to the school/children? *Type answer below.*

**Q7:** Which aspects of the project did you like least and why? If you have any changes you would recommend if were to duplicate this study, please tell use here. *Type answer below.*

**Q8:** Which aspects of the project did you like most and why? *Type answer below.*

**Q9:** Would you be interested in participating in a similar project in the autumn/winter? *Type answer below.*

**LONG-TERM FOLLOW-UP QUESTIONNAIRE FOR TEACHERS**

**Q1:** Name of school *[short answer text here].*

**Q2:** Your name *[short answer text here]*

**Q3a:** Is your school still feeding birds? *[Tick appropriate box]*

- Yes – all year round (please go to question 3b)
- Yes – sometimes (please go to question 3b)
- No (please go to question 3c)

**Q3b:** Please provide any further information on this; e.g. how often, what time of year, who does the feeding, have any additional bird feeding materials been bought? *[Type answer below]*

**Q3c:** What are the main reasons for this? *[Type answer below]*

**Q4a:** Have any of the following parts of the Bird Buddies project been repeated with the same and/or other class(es)? *[Tick appropriate boxes]*

- Bird watching in school grounds
- Bird surveys in school grounds
- Using the games/education resources provided
- None

**Q4b:** Please write any comments to the above question here. *[Type answer below]*

**Q5a:** Has the class/school engaged in any other environmental project/activities as a result of Bird Buddies? *[Tick appropriate box]*

- Yes
- No

**Q5b:** If yes, please briefly state what these have been. *[Type answer below]*

**Q6:** If you have any final comments, please include them here. *[Type answer below]*
