# Supplementary material for: Birds in the playground: Evaluating the effectiveness of an urban environmental education project in enhancing school children’s awareness, knowledge and attitudes towards local wildlife
Source: PLoS One. 2018 Mar 6;13(3):e0193993. doi: 10.1371/journal.pone.0193993 (PMC5839573; doi:10.1371/journal.pone.0193993)
Supplement: S5 Appendix — Summary statistics for sociodemographic attributes of participants (Table A). Species included in identification test and seen during bird surveys (Table B). Results of paired Wilcoxon signed rank tests between pre- and post-project bird identification scores per sociodemographic factor (Table C). Percentage of children at each school and year group that correctly identified bird species pre-project and post-project (Table D). Species misidentifications in pre- and post-project tests (Table E). Number of species misidentifications and blank answers in pre- and post-project tests per species (Table F). Results of paired Wilcoxon signed rank tests between pre- and post-project composite, affect and utility attitude scores per sociodemographic factor (Table G). Descriptive statistics for pre- and post-project attitude scores per sociodemographic factor (Table H). Results of Mann-Whitney or Kruskal-Wallis tests per sociodemographic factor for pre- and post-project attitude scores and change in score (Table I). Proportion of children per school that scored either “Agree” or “Strongly agree” for student evaluation statements (Table J). (DOCX) [file pone.0193993.s005.docx]

**S5 Appendix:** Supplementary results

**Table A. Summary statistics for sociodemographic attributes of participants.**

| **Variable** | **Summary statistics** | |
| --- | --- | --- |
| **School (8 levels)** |  |  |
| A | 14.5 % | (n = 32) |
| B | 8.2 % | (n = 18) |
| C | 14.1 % | (n = 31) |
| D | 10.0 % | (n = 22) |
| E | 14.1 % | (n = 31) |
| F | 11.0 % | (n = 24) |
| G | 14.5 % | (n = 32) |
| H | 13.6 % | (n = 30) |
| **School year (3 levels)** |  |  |
| Year 3 | 21.8 % | (n = 48) |
| Year 4 | 29.1 % | (n = 64) |
| Year 5 | 49.1 % | (n = 108) |
| **Gender (2 levels)** |  |  |
| Male | 58.6 % | (n = 129) |
| Female | 41.4 % | (n = 91) |
| **Outdoor space at home* (2/3 levels)** |  |  |
| Yes, with grass | 70.6 % | (n = 154) |
| Yes, without grass | 19.3 % | (n = 42) |
| None | 10.1 % | (n = 22) |
| **Birds seen: home outdoor space* (3 levels)** | |  |
| Yes | 82.6 % | (n = 180) |
| No | 7.3 % | (n = 16) |
| No outdoor space | 10.1 % | (n = 22) |
| **Family feed birds: home outdoor space* (3/4 levels)** | |  |
| Yes, all year round | 15.1 % | (n = 33) |
| Yes, sometimes | 34.9 % | (n = 76) |
| No | 39.9 % | (n = 87) |
| No outdoor space | 10.1 % | (n = 22) |
| **Pets* (2 levels)** |  |  |
| Yes | 65.1 % | (n = 142) |
| No | 34.9 % | (n = 76) |

* Total sample size equals 218 respondents due to two missing the pre-project questionnaire.

**Table B. Species included in identification test and seen at least once during bird surveys per school (indicated by an X).** Species underlined are those included in the project identification tests.

| **Species/School** | **A** | **B** | **C** | **D** | **E** | **F** | **G** | **H** |
| --- | --- | --- | --- | --- | --- | --- | --- | --- |
| **Blue tit** |  | X | X |  | X | X | X | X |
| **Robin** | X | X | X |  | X | X | X | X |
| **House sparrow** | X | X | X | X | X | X | X |  |
| **Chaffinch** |  |  |  |  | X |  |  |  |
| **Greenfinch** |  |  |  |  | X |  |  |  |
| **Wren** | X |  | X | X | X |  | X | X |
| **Blackbird** | X | X | X | X | X | X | X |  |
| **Starling** | X | X | X | X | X | X | X | X |
| **Magpie** | X | X | X | X | X | X | X | X |
| **Collared dove** |  | X | X |  | X | X | X |  |
| **(Carrion) crow** | X |  | X | X | X |  | X | X |
| **Black-headed gull** | X | X | X | X | X |  |  |  |
| **SUBTOTAL** | 8 | 8 | 10 | 7 | 12 | 7 | 9 | 6 |
| **Song thrush** |  | X | X | X |  |  | X |  |
| **Coal tit** | X | X | X |  | X | X | X |  |
| **Long-tailed tit** |  | X |  |  |  |  | X |  |
| **Dunnock** | X |  |  |  | X |  | X |  |
| **Pied wagtail** | X | X |  |  |  |  | X |  |
| **Jackdaw** | X | X |  | X | X |  | X |  |
| **Woodpigeon** | X | X | X | X | X | X | X | X |
| **Great tit** |  |  | X |  |  | X | X | X |
| **Herring gull** | X | X | X | X | X |  | X |  |
| **TOTAL** | **14** | **15** | **15** | **11** | **17** | **10** | **18** | **10** |

**Table C. Results of paired Wilcoxon signed rank tests between pre- and post-project bird identification scores per sociodemographic factor.** Increase in scores are shown as a percentage. For the factor “Feed birds”, the levels “yes – all year round” and “yes – sometimes” have been merged.

|  |  |  | Pre-project score | | Post-project score | | Wilcoxon signed rank test | | Score increase |
| --- | --- | --- | --- | --- | --- | --- | --- | --- | --- |
|  |  | ***n*** | **Mean (+/-SD)** | **Min /Max.** | **Mean (+/-SD)** | **Min /Max** | **w** | ***p*** | **%** |
| *School* | A | 31 | 11.3 (5.0) | 3 / 24 | 15.7 (6.2) | 4 / 24 | 83 | **0.006** | 19 |
|  | B | 16 | 5.6 (4.6) | 0 / 14 | 18.3 (6.2) | 3 / 24 | 0.0 | **<0.001** | 53 |
|  | C | 30 | 13.2 (6.0) | 2 / 23 | 19.8 (6.0) | 4 / 24 | 6.0 | **<0.001** | 28 |
|  | D | 21 | 6.3 (3.8) | 0 / 15 | 12.9 (4.7) | 5 / 22 | 1.0 | **<0.001** | 27 |
|  | E | 26 | 9.8( 2.9) | 0 / 14 | 13.4 (4.1) | 3 / 19 | 22.0 | **<0.001** | 15 |
|  | F | 19 | 6.9 (4.4) | 0 / 12 | 12.6 (4.6) | 2 / 19 | 5.5 | **<0.001** | 24 |
|  | G | 30 | 6.1 (2.9) | 0 / 13 | 16.4 (5.4) | 6 / 24 | 1.0 | **<0.001** | 43 |
|  | H | 29 | 7.6 (3.2) | 0 / 15 | 20.6 (3.4) | 11 / 24 | 0.0 | **<0.001** | 54 |
| *Gender* | Male | 119 | 7.9 (4.4) | 0 / 23 | 16.2 (6.0) | 3 / 24 | 176 | **<0.001** | 35 |
|  | Female | 83 | 9.9 (5.4) | 0 / 24 | 16.8 (5.7) | 2 / 24 | 135.5 | **<0.001** | 29 |
| *School year* | Year 3 | 45 | 6.8 (3.9) | 0 / 15 | 19.8 (4.7) | 3 / 24 | 0.0 | **<0.001** | 54 |
|  | Year 4 | 61 | 8.8 (4.9) | 0 / 24 | 16.0 (5.8) | 4 / 24 | 130 | **<0.001** | 30 |
|  | Year 5 | 96 | 9.5 (5.3) | 0 / 23 | 15.1 (5.9) | 2 / 24 | 100 | **<0.001** | 23 |
| Outdoor space | Grass | 141 | 8.5 (4.8) | 0 / 24 | 16.1 (5.8) | 2 / 24 | 407 | **<0.001** | 32 |
|  | No grass | 40 | 8.5 (5.0) | 0 / 23 | 17.5 (5.7) | 3 / 24 | 7.5 | **<0.001** | 38 |
|  | None | 21 | 10.4 (5.6) | 2 / 23 | 16.7 (6.4) | 4 / 24 | 5.5 | **<0.001** | 26 |
| *Birds seen* | Yes | 167 | 8.7 (5.0) | 0 / 24 | 16.2 (5.7) | 2 / 24 | 495 | **<0.001** | 32 |
| *(outdoor space)* | No | 14 | 6.6 (2.7) | 0 /10 | 18.9 (6.4) | 6 / 24 | 0.0 | **0.002** | 50 |
| *Feed birds* | Yes | 101 | 9.5 (5.1) | 0 / 24 | 16.4 (5.7) | 2 / 24 | 256 | **<0.001** | 29 |
| *(outdoor space)* | No | 89 | 7.3 (4.3) | 0 / 23 | 16.5 (6.0) | 3 / 24 | 40.5 | **<0.001** | 38 |
| *Pets* | Yes | 131 | 9.0 (4.9) | 0 / 23 | 16.5 (5.6) | 3 / 24 | 263 | **<0.001** | 31 |
|  | No | 71 | 8.1 (5.0) | 0 /24 | 16.4 (6.4) | 2 / 24 | 71.5 | **<0.001** | 35 |

**Table D.** **Percentage of children at each school and year group that correctly identified bird species (i.e. scored 2 points): (A) pre-project and (B) post-project.** Correct identification for each species across all children and overall average for each group are also shown. Year group and species ranked highest (1) to lowest (3/12). Highest values (pre- vs. post-project) are shaded in green.

| **A)** |  |  |  |  |  |  |  |  |  |  |  |  |  |  |
| --- | --- | --- | --- | --- | --- | --- | --- | --- | --- | --- | --- | --- | --- | --- |
|  | **Average correct % (rank)** | ***N*** | **Blue tit** | **Robin** | **House sparrow** | **Chaffinch** | **Greenfinch** | **Wren** | **Blackbird** | **Starling** | **Magpie** | **Collared dove** | **(Carrion) crow** | **Black-headed gull** |
| **School A** | 42.5 (2) | 31 | 77.4 | 100.0 | 3.2 | 22.6 | 9.7 | 9.7 | 64.5 | 19.4 | 80.6 | 12.9 | 83.9 | 25.8 |
| **School B** | 18.2 (8) | 16 | 6.3 | 75.0 | 0.0 | 25.0 | 0.0 | 0.0 | 31.3 | 0.0 | 37.5 | 0.0 | 43.8 | 0.0 |
| **School C** | 52.2 (1) | 30 | 73.3 | 86.7 | 16.7 | 50.0 | 43.3 | 33.3 | 53.3 | 50.0 | 50.0 | 10.0 | 66.7 | 93.3 |
| **School D** | 23.0 (6) | 21 | 28.6 | 57.1 | 0.0 | 4.8 | 0.0 | 0.0 | 19.0 | 9.5 | 71.4 | 0.0 | 80.9 | 4.8 |
| **School E** | 35.2 (3) | 26 | 76.9 | 96.2 | 6.5 | 0.0 | 0.0 | 11.5 | 57.7 | 0.0 | 96.2 | 0.0 | 61.5 | 15.4 |
| **School F** | 24.1 (5) | 19 | 36.8 | 73.7 | 0.0 | 0.0 | 0.0 | 10.5 | 52.6 | 5.3 | 57.9 | 0.0 | 52.6 | 0.0 |
| **School G** | 22.2 (7) | 30 | 13.3 | 96.7 | 0.0 | 0.0 | 0.0 | 3.3 | 40.0 | 0.0 | 56.7 | 3.3 | 53.3 | 0.0 |
| **School H** | 26.7 (4) | 29 | 55.1 | 93.1 | 0.0 | 0.0 | 0.0 | 0.0 | 27.6 | 17.2 | 72.4 | 0.0 | 55.1 | 0.0 |
| **Year 3** | 23.7 (3) | 45 | 37.8 | 86.7 | 0.0 | 8.9 | 0.0 | 0.0 | 28.9 | 11.1 | 60.0 | 0.0 | 51.1 | 0.0 |
| **Year 4** | 32.5 (2) | 61 | 45.9 | 98.4 | 1.6 | 11.5 | 4.9 | 6.6 | 52.5 | 9.8 | 68.9 | 8.2 | 68.9 | 13.1 |
| **Year 5** | 35.6 (1) | 96 | 57.3 | 80.2 | 6.3 | 16.7 | 13.5 | 15.6 | 46.9 | 18.8 | 68.8 | 3.1 | 65.6 | 34.4 |
| **OVERALL** | **32.0** | **202** | **49.5** | **87.1** | **3.5** | **13.4** | **7.9** | **9.4** | **44.6** | **14.4** | **66.8** | **4.0** | **63.4** | **20.3** |
| **Rank** |  |  | **4** | **1** | **12** | **8** | **10** | **9** | **5** | **7** | **2** | **11** | **3** | **6** |
| **B)** |  |  |  |  |  |  |  |  |  |  |  |  |  |  |
| **School A** | 63.2 (5) | 31 | 80.6 | 90.3 | 80.6 | 35.5 | 25.8 | 67.7 | 83.9 | 32.3 | 96.8 | 41.9 | 58.1 | 64.5 |
| **School B** | 74.0 (3) | 16 | 93.8 | 87.5 | 75.0 | 81.3 | 62.5 | 43.8 | 100 | 75.0 | 87.5 | 31.3 | 68.8 | 81.3 |
| **School C** | 81.4 (2) | 30 | 86.7 | 93.3 | 60.0 | 76.7 | 76.7 | 73.3 | 86.7 | 86.7 | 90.0 | 70.0 | 86.7 | 90.3 |
| **School D** | 49.2 (7) | 21 | 81.0 | 100 | 9.5 | 19.0 | 28.6 | 0.0 | 71.4 | 57.1 | 81.0 | 19.0 | 85.7 | 38.1 |
| **School E** | 50.6 (6) | 26 | 96.2 | 96.2 | 7.7 | 3.8 | 26.9 | 19.2 | 61.5 | 57.7 | 96.2 | 3.8 | 84.6 | 53.8 |
| **School F** | 47.8 (8) | 19 | 73.7 | 89.5 | 47.3 | 15.8 | 42.1 | 0.0 | 68.4 | 57.9 | 84.2 | 10.5 | 68.4 | 15.8 |
| **School G** | 64.7 (4) | 30 | 90.0 | 93.3 | 63.3 | 26.7 | 30.0 | 43.3 | 83.3 | 73.3 | 83.3 | 43.3 | 80.0 | 66.7 |
| **School H** | 82.2 (1) | 29 | 93.1 | 100 | 17.2 | 75.9 | 89.7 | 75.9 | 89.7 | 96.6 | 96.6 | 69.0 | 93.1 | 89.7 |
| **Year 3** | 78.5 (1) | 45 | 91.1 | 95.6 | 37.8 | 75.6 | 80.0 | 64.4 | 93.3 | 88.9 | 93.3 | 51.1 | 84.4 | 86.7 |
| **Year 4** | 63.9 (2) | 61 | 85.2 | 91.8 | 72.1 | 31.1 | 27.9 | 55.7 | 83.6 | 52.5 | 90.2 | 42.6 | 68.9 | 65.6 |
| **Year 5** | 59.3 (3) | 96 | 85.4 | 94.8 | 32.3 | 32.3 | 45.8 | 28.1 | 74.0 | 66.7 | 88.5 | 26.0 | 82.3 | 55.2 |
| **OVERALL** | **65.0** | **202** | **87.1** | **94.1** | **45.5** | **41.6** | **48.0** | **44.6** | **81.2** | **67.3** | **90.1** | **36.6** | **78.7** | **65.3** |
| **Rank** |  |  | **3** | **1** | **8** | **11** | **9** | **10** | **4** | **6** | **2** | **12** | **5** | **7** |

**Table E. Species misidentifications in pre- and post-project tests.** Names in *italics* indicate non-native birds, names in **bold** indicate “made-up” birds and names underlined indicate partially correct identification. Numbers equal the frequency with which each name was provided as an answer. Names are ordered alphabetically.

| **Blue tit** | | | | **Robin** | | | |
| --- | --- | --- | --- | --- | --- | --- | --- |
| Pre-project | | Post-project | | Pre-project | | Post-project | |
| **Blue finch** | 1 | Coal tit | 1 | Chaffinch | 1 | Chaffinch | 1 |
| **Blue great tit** | 1 | Great tit | 7 |  |  | **Riding** | 1 |
| *Blue jay* | 3 | Long-tailed tit | 1 |  |  | Tit | 1 |
| Blue | 3 | Sparrow | 1 |  |  |  |  |
| *Bluebird* | 4 | Tit | 1 |  |  |  |  |
| *Budgie* | 2 |  |  |  |  |  |  |
| Kingfisher | 8 |  |  |  |  |  |  |
| Lark | 1 |  |  |  |  |  |  |
| Magpie | 1 |  |  |  |  |  |  |
| *Parrot* | 4 |  |  |  |  |  |  |
| Puffin | 1 |  |  |  |  |  |  |
| **Greenfinch** | | | | **Wren** | | | |
| Pre-project | | Post-project | | Pre-project | | Post-project | |
| *Budgie* | 2 | Chaffinch | 2 | *Chickadee* | 3 | (House) sparrow | 15 |
| Chaffinch | 1 | Coal tit | 1 | House martin | 1 | (Song) thrush | 6 |
| Goldfinch | 2 | **Gold dove** | 1 | *Hummingbird* | 3 | Dove | 1 |
| Great tit | 7 | Goldfinch | 4 | **Jack sparrow** | 1 | Dunnock | 5 |
| **Green bird** | 1 | Great tit | 5 | Jackdaw | 1 | Finch | 2 |
| **Green guggler** | 1 | **Green tit** | 6 | Kingfisher | 1 | Goldfinch | 2 |
| **Green jay** | 1 | Wren | 1 | Long tailed tit | 2 | Great tit | 1 |
| **Green tit** | 2 | **Yellow tit** | 2 | Marsh tit | 1 | **Long-tailed finch** | 1 |
| *Parakeet* | 1 | **Yellow-tailed tit** | 1 | Pigeon | 1 | Quail | 1 |
| Robin | 1 |  |  | Robin | 2 | Song wren | 1 |
| Swallow | 4 |  |  | Sparrow | 19 | Starling | 3 |
| **Yellow tit** | 1 |  |  | Starling | 2 | Wagtail | 3 |
|  |  |  |  | Swallow | 1 | Woodpigeon | 1 |
|  |  |  |  | Thrush | 2 | Wren chaffinch | 1 |
|  |  |  |  | Woodpecker | 5 |  |  |
| **House sparrow** | | | | **Chaffinch** | | | |
| Pre-project | | Post-project | | Pre-project | | Post-project | |
| Chaffinch | 3 | Bullfinch | 2 | Long-tailed tit | 1 | Great tit | 5 |
| Finch | 2 | Chaffinch | 4 | **Red-bellied robin** | 2 | Jay | 1 |
| Great tit | 3 | Coal tit | 5 | **Red jay** | 7 | **Red jay** | **6** |
| Heron | 2 | Dunnock | 1 | Robin | 1 | **Red tit** | **2** |
| Jackdaw | 2 | Finch | 1 | Skylark | 3 | Skylark | 4 |
| Kingfisher | 1 | Great tit | 10 | Woodpecker | 2 | Song thrush | 2 |
| *Kookaburra* | 1 | Long-tailed tit | 2 |  |  | Sparrow | 1 |
| Sparrow | 8 | Robin | 1 |  |  | Wood pigeon | 1 |
| Sparrowhawk | 1 | Song thrush | 3 |  |  |  |  |
| Starling | 2 | Sparrow | 25 |  |  |  |  |
| Tit | 1 | Woodpecker | 4 |  |  |  |  |
| Woodpecker | 1 | Wren | 1 |  |  |  |  |
| Wren | 5 |  |  |  |  |  |  |

**Table E. Continued.**

| **Blackbird** | | | | **Starling** | | | |
| --- | --- | --- | --- | --- | --- | --- | --- |
| Pre-project | | Post-project | | Pre-project | | Post-project | |
| Badger | 1 | **Black tit** | 1 | Coal tit | 1 | Coal tit | 1 |
| **Beaked crow** | 1 | Crow | 7 | Greenfinch | 1 | Swallow | 1 |
| Black… | 1 | Jackdaw | 1 | *Hummingbird* | 1 | Thrush | 2 |
| (Carrion) crow | 24 | Raven | 1 | Kingfisher | 13 | Woodpecker | 3 |
| **Dark warbler** | 1 |  |  | *Kiwi* | 1 |  |  |
| Jackdaw | 1 |  |  | Magpie | 1 |  |  |
| Magpie | 3 |  |  | Nightingale | 2 |  |  |
| Pigeon | 1 |  |  | Pheasant | 1 |  |  |
| Rook | 1 |  |  | Thrush | 3 |  |  |
| Sparrow | 4 |  |  | Woodpecker | 10 |  |  |
| Starling | 1 |  |  |  |  |  |  |
| **Magpie** | | | | **Collared dove** | | | |
| Pre-project | | Post-project | | Pre-project | | Post-project | |
| Blackbird | 1 | Black headed | 1 | Dove | 15 | **Black cole dove** | 1 |
| *Bluebird* | 1 |  |  | Dove/pigeon | 3 | **Black-collared dove** | 3 |
| Pigeon | 2 |  |  | Duck | 1 | Coal tit | 1 |
| Sparrow | 3 |  |  | Pheasant | 1 | **Colder dove** | 1 |
| Wagtail | 1 |  |  | Pigeon | 40 | **Coloured dove** | 5 |
| Woodpecker | 1 |  |  | Sparrow | 1 | **Corn dove** | 1 |
|  |  |  |  | Starling | 1 | Dove | *18* |
|  |  |  |  | Turtle dove | 2 | Duck | 1 |
|  |  |  |  | **White pigeon** | 1 | Dunnock | 1 |
|  |  |  |  | Wood pigeon | 10 | Great tit |  |
|  |  |  |  |  |  | Sparrow |  |
|  |  |  |  |  |  | Starling |  |
|  |  |  |  |  |  | (Wood) pigeon | **36** |
| **(Carrion) crow** | | | | **Black-headed gull** | | | |
| Pre-project | | Post-project | | Pre-project | | Post-project | |
| **Black kind** | 1 | Blackbird | 8 | **Black-eyed gull** | 1 | Black-eyed seagull | 1 |
| Blackbird | 15 | Dunnock | 1 | Dove | 2 | Herring gull | 24 |
| Jackdaw | 3 | Jackdaw | 4 | Duck | 1 | Pigeon | 1 |
| Raven | 14 | Magpie | 1 | Kingfisher | 2 | (Sea)gull | 22 |
| Rook | 1 | Raven | 3 | Pigeon | 2 | White-headed gull | 2 |
| Sparrow | 4 | Starling | 1 | (Sea)gull | 97 |  |  |
| Woodpecker | 1 |  |  | **South born seagull** | 1 |  |  |
| Wren | 1 |  |  |  |  |  |  |

**Table F. Number of species misidentifications and blank answers in pre- and post-project tests per species.** Incorrect answers scored zero and partially correct answers scored one point in the species identification tests. Highest values are underlined to ease pre- versus post-project comparison.

|  | **Pre-project** | | |  | **Post-project** | | |
| --- | --- | --- | --- | --- | --- | --- | --- |
|  | **Incorrect** | **Partially correct** | **Unanswered** |  | **Incorrect** | **Partially correct** | **Unanswered** |
| **Blue tit** | 28 | 1 | 73 |  | 1 | 9 | 14 |
| **Robin** | 1 | 0 | 25 |  | 3 | 0 | 8 |
| **House sparrow** | 24 | 8 | 163 |  | 34 | 25 | 51 |
| **Chaffinch** | 16 | 0 | 159 |  | 22 | 0 | 97 |
| **Greenfinch** | 21 | 3 | 162 |  | 17 | 6 | 78 |
| **Wren** | 45 | 0 | 138 |  | 41 | 2 | 68 |
| **Blackbird** | 39 | 0 | 73 |  | 10 | 0 | 27 |
| **Starling** | 34 | 0 | 139 |  | 7 | 0 | 58 |
| **Magpie** | 9 | 0 | 58 |  | 1 | 0 | 19 |
| **Collared dove** | 6 | 69 | 119 |  | 7 | 65 | 54 |
| **(Carron) crow** | 22 | 18 | 34 |  | 11 | 7 | 24 |
| **Black-headed gull** | 7 | 99 | 46 |  | 1 | 49 | 16 |
| **TOTAL** | **252** | **198** | **1189** |  | **155** | **163** | **514** |

**Table G. Results of paired Wilcoxon signed rank tests between pre- and post-project composite, affect and utility attitude scores per sociodemographic factor.** Increase in scores are shown as a percentage. For the factor “Feed birds”, the levels “yes – all year round” and “yes – sometimes” have been merged. See Table H for corresponding descriptive statistics.

|  |  | Composite | | | | Affect | | | Utility | | |
| --- | --- | --- | --- | --- | --- | --- | --- | --- | --- | --- | --- |
|  |  | ***n*** | **w** | ***p*** | **%** | **w** | ***p*** | **%** | **w** | ***p*** | **%** |
| *School* | A | 30 | 40 | **<0.001** | 10 | 0.0 | **0.048** | 5 | 46 | **0.001** | 14 |
|  | B | 14 | 6.0 | **0.009** | 13 | 0.0 | **0.037** | 8 | 15 | **0.029** | 17 |
|  | C | 24 | 49.5 | 0.120 | -3 | 0.0 | 1.000 | 1 | 60 | 0.777 | -6 |
|  | D | 18 | 52.5 | 1.000 | -4 | 3.0 | 1.000 | 0 | 55 | 0.894 | -4 |
|  | E | 22 | 10.0 | **0.011** | 7 | 1.0 | 1.000 | -1 | 4.5 | **0.006** | 15 |
|  | F | 16 | 12.0 | 0.212 | 5 | 6.0 | 0.850 | -1 | 9 | **0.052** | 11 |
|  | G | 20 | 15.0 | 0.183 | 3 | 0.0 | 0.149 | 3 | 23 | 0.666 | 2 |
|  | H | 23 | 16.0 | **0.007** | 14 | 6.0 | 0.098 | 8 | 20 | **0.012** | 18 |
| *Gender* | Male | 69 | 655 | **0.005** | 5 | 41.0 | **0.040** | 2 | 756 | **0.019** | 6 |
|  | Female | 98 | 149.5 | **<0.001** | 9 | 15.0 | 0.053 | 4 | 169 | **<0.001** | 13 |
| *School year* | Year 3 | 37 | 39 | **<0.001** | 14 | 11.0 | **0.012** | 8 | 66.5 | **<0.001** | 18 |
|  | Year 4 | 50 | 101.5 | **<0.001** | 7 | 0.0 | **0.008** | 4 | 140 | **0.004** | 10 |
|  | Year 5 | 80 | 440.5 | 0.172 | 2 | 25.0 | 0.803 | 0 | 439.5 | 0.105 | 4 |
| Outdoor space | Grass | 119 | 510 | **<0.001** | 8 | 43.5 | **0.009** | 3 | 602 | **<0.001** | 11 |
|  | No grass | 35 | 130 | 0.371 | 3 | 10.5 | 0.588 | 1 | 138.5 | 0.500 | 4 |
|  | None | 13 | 16 | 0.454 | 4 | 0.0 | 0.346 | 3 | 22.5 | 0.627 | 3 |
| *Birds seen* | Yes | 143 | 1054 | **<0.001** | 6 | 14.5 | **0.051** | 3 | 1171.5 | **<0.001** | **9** |
| *(outdoor space)* | No | 11 | 4 | 0.056 | 13 | 0.0 | 0.089 | 10 | 6.0 | 0.196 | 14 |
| *Feed birds* | Yes | 86 | 228 | **<0.001** | 6 | 14.5 | **0.051** | 3 | 289 | **<0.001** | 9 |
| *(outdoor space)* | No | 68 | 357.5 | **0.003** | 7 | 37.5 | 0.092 | 3 | 372 | **0.008** | 10 |
| *Pets* | Yes | 107 | 541.5 | **<0.001** | 7 | 37.0 | **0.002** | 4 | 690.5 | **<0.001** | 10 |
|  | No | 60 | 217 | **0.038** | 4 | 16.0 | 0.821 | 0 | 221.5 | **0.041** | 8 |

**Table H. Descriptive statistics (mean +/- SD) for pre- and post-project attitude scores (composite, affect, and utility) per sociodemographic factor.**

|  |  |  | Composite | | Affect | | Utility | |
| --- | --- | --- | --- | --- | --- | --- | --- | --- |
|  |  | ***n*** | **Pre** | **Post** | **Pre** | **Post** | **Pre** | **Post** |
| *School* | A | 30 | 5.5 (2.2) | 7.2 (1.2) | 3.5 (1.3) | 4.0 (0.0) | 1.9 (1.4) | 3.2 (1.2) |
|  | B | 14 | 4.4 (1.9) | 6.7 (1.5) | 3.3 (1.0) | 4.0 (0.0) | 1.1 (1.5) | 2.7 (1.5) |
|  | C | 24 | 7.3 (1.2) | 6.8 (1.5) | 3.9 (0.4) | 4.0 (0.0) | 3.3 (1.1) | 2.8 (1.5) |
|  | D | 18 | 4.3 (3.4) | 4.3 (3.7) | 3.0 (1.8) | 3.0 (1.7) | 1.3 (1.9) | 1.3 (2.5) |
|  | E | 22 | 5.4 (2.3) | 6.6 (2.3) | 3.8 (0.6) | 3.7 (0.9) | 1.5 (2.04) | 2.9 (1.6) |
|  | F | 16 | 5.8 (1.9) | 6.6 (1.9) | 3.8 (0.7) | 3.6 (1.1) | 2.0 (1.5) | 3.0 (1.5) |
|  | G | 20 | 6.1 (1.7) | 6.6 (1.6) | 3.6 (1.0) | 3.9 (0.44) | 2.5 (1.4) | 2.7 (1.3) |
|  | H | 23 | 4.2 (3.5) | 6.5 (1.6) | 3.0 (1.8) | 3.7 (0.7) | 1.1 (2.7) | 2.8 (1.3) |
| *Gender* | Male | 69 | 5.5 (2.5) | 6.3 (2.3) | 3.5 (1.1) | 3.8 (0.9) | 2.0 (1.9) | 2.6 (1.7) |
|  | Female | 98 | 5.3 (2.7) | 6.7 (1.8) | 3.5 (1.4) | 3.8 (0.8) | 1.8 (1.8) | 2.9 (1.4) |
| *School year* | Year 3 | 37 | 4.3 (3.0) | 6.6 (1.6) | 3.1 (1.5) | 3.9 (0.6) | 1.1 (2.3) | 2.8 (1.4) |
|  | Year 4 | 50 | 5.7 (2.0) | 7.0 (1.4) | 3.6 (1.2) | 4.0 (0.3) | 2.2 (1.4) | 3.0 (1.3) |
|  | Year 5 | 80 | 5.8 (2.5) | 6.2 (2.6) | 3.7 (1.0) | 3.6 (1.1) | 2.1 (1.8) | 2.5 (1.9) |
| Outdoor space | Grass | 119 | 5.5 (2.4) | 6.8 (1.6) | 3.5 (1.2) | 3.8 (0.6) | 1.9 (1.8) | 2.9 (1.4) |
|  | No grass | 35 | 5.3 (3.0) | 5.8 (3.0) | 3.4 (1.3) | 3.5 (1.3) | 1.8 (2.1) | 2.2 (2.0) |
|  | None | 13 | 5.4 (2.6) | 6.0 (2.7) | 3.4 (1.3) | 3.7 (1.1) | 2.0 (2.0) | 2.3 (1.8) |
| *Birds seen* | Yes | 143 | 5.5 (2.6) | 6.6 (2.1) | 3.6 (1.2) | 3.8 (0.8) | 2.0 (1.8) | 2.8 (1.6) |
| *(outdoor space)* | No | 11 | 4.0 (2.2) | 6.2 (1.9) | 2.9 (1.6) | 3.8 (0.6) | 1.1 (2.3) | 2.4 (1.5) |
| *Feed birds* | Yes | 86 | 5.8 (2.5) | 6.8 (1.7) | 3.5 (1.3) | 3.8 (0.8) | 2.2 (1.6) | 3.0 (1.3) |
| *(outdoor space)* | No | 68 | 5.0 (2.6) | 6.2 (2.4) | 3.5 (1.1) | 3.8 (0.9) | 1.5 (2.1) | 2.4 (1.8) |
| *Pets* | Yes | 107 | 5.4 (2.4) | 6.7 (2.8) | 3.5 (1.2) | 3.9 (0.6) | 2.0 (1.7) | 2.8 (1.4) |
|  | No | 60 | 5.4 (2.8) | 6.1 (1.6) | 3.6 (1.2) | 3.6 (1.2) | 1.8 (2.2) | 2.5 (1.9) |
| OVERALL |  | 167 | 5.4 (2.6) | 6.5 (2.1) | 3.5 (1.2) | 3.8 (0.8) | 1.9 (1.9) | 2.7 (1.6) |

**Table I. Results of Mann-Whitney (MW) or Kruskall-Wallis (KW) tests per sociodemographic factor for pre- and post-project attitude scores and change in score (composite, affect and utility).** For sample sizes see Table H.

|  |  |  |  | Pre-project score | | Post-project score | | Change in score* | |
| --- | --- | --- | --- | --- | --- | --- | --- | --- | --- |
|  |  | T**est** | **Levels** | **value** | ***p*** | **value** | ***p*** | **value** | ***p*** |
| *School* | Composite | KW | 8 | 26.2 | **<0.001** | 12.1 | 0.100 | 22.1 | **0.002** |
|  | Affect | KW | 8 | 10.5 | 0.160 | 17.9 | **0.012** | 11.9 | 0.104 |
|  | Utility | KW | 8 | 26.0 | **<0.001** | 11.1 | 0.133 | 22.4 | **0.002** |
| *Gender* | Composite | MW | 2 | 3202 | 0.541 | 3704 | 0.300 | 3957.5 | **0.043** |
|  | Affect | MW | 2 | 3414 | 1.000 | 3414 | 0.820 | 3427 | 0.825 |
|  | Utility | MW | 2 | 3124.5 | 0.373 | 3.723.5 | 0.218 | 3920 | 0.068 |
| *School year* | Composite | KW | 3 | 9.1 | **0.011** | 11.1 | 0.259 | 11.1 | **0.004** |
|  | Affect | KW | 3 | 5.4 | 0.067 | 3.8 | 0.147 | 9.9 | **0.007** |
|  | Utility | KW | 3 | 6.0 | 0.051 | 1.6 | 0.450 | 6.9 | **0.032** |
| *Outdoor space* | Composite | KW | 3 | 0.1 | 0.999 | 3.3 | 0.191 | 3.65 | 0.161 |
|  | Affect | KW | 3 | 0.6 | 0.739 | 1.1 | 0.584 | 0.2 | 0.885 |
|  | Utility | KW | 3 | 0.1 | 0.982 | 4.2 | 0.124 | 3.3 | 0.194 |
| *Birds seen* | Composite | MW | 2 | 468.5 | **0.019** | 658.5 | 0.325 | 951 | 0.234 |
| *(outdoor space)* | Affect | MW | 2 | 614.5 | 0.065 | 778 | 0.904 | 996.5 | **0.029** |
|  | Utility | MW | 2 | 613.5 | 0.195 | 638.5 | 0.250 | 862 | 0.583 |
| *Feed birds* | Composite | MW | 2 | 2387 | **0.040** | 2511.5 | 0.100 | 3011 | 0.745 |
| *(outdoor space)* | Affect | MW | 2 | 2759 | 0.358 | 2941.5 | 0.894 | 2993 | 0.710 |
|  | Utility | MW | 2 | 2378 | **0.034** | 2430 | **0.046** | 2951.5 | 0.918 |
| *Pets* | Composite | MW | 2 | 3329 | 0.677 | 3038 | 0.530 | 2911.5 | 0.304 |
|  | Affect | MW | 2 | 3391.5 | 0.359 | 3004 | 0.140 | 2848 | 0.071 |
|  | Utility | MW | 2 | 3254 | 0.877 | 3045.5 | 0.545 | 2958 | 0.382 |

* Due to normality of data, parametric tests were instead applied, specifically two-sample t-test or one-way ANOVA.

**Table J. Proportion of children per school (A-H) that scored either “Agree” or “Strongly agree” for the nine statements comprising the *student evaluation score.***

| Statement | A (n = 32) | B (n = 16) | C (n = 30) | D (n = 20) | E (n = 26) | F (n = 19) | G (n = 29) | H (n = 29) | OVERALL (201) |
| --- | --- | --- | --- | --- | --- | --- | --- | --- | --- |
| This project has improved my bird knowledge | 84 | 75 | 93 | 70 | 81 | 79 | 97 | 100 | 87 |
| This project has improved my science skills | 69 | 56 | 67 | 40 | 42 | 58 | 45 | 72 | 57 |
| I want to continue feeding birds at school | 100 | 100 | 90 | 65 | 85 | 100 | 97 | 90 | 91 |
| I want to continue surveying birds at school | 97 | 94 | 80 | 55 | 46 | 100 | 90 | 83 | 81 |
| I want to continue learning about local wildlife | 100 | 81 | 87 | 80 | 77 | 74 | 90 | 90 | 86 |
| Feeding birds has helped increase the number of birds in our school grounds | 75 | 75 | 77 | 80 | 81 | 100 | 86 | 83 | 82 |
| I am more likely to go birdwatching | 72 | 88 | 50 | 50 | 46 | 68 | 93 | 86 | 69 |
| I am more likely to read about birds | 81 | 69 | 75 | 30 | 27 | 53 | 66 | 76 | 58 |
| I am more likely to watch TV shows on birds | 81 | 50 | 43 | 40 | 23 | 58 | 34 | 66 | 50 |
