# Supplementary material for: Birds in the playground: Evaluating the effectiveness of an urban environmental education project in enhancing school children’s awareness, knowledge and attitudes towards local wildlife
Source: PLoS One. 2018 Mar 6;13(3):e0193993. doi: 10.1371/journal.pone.0193993 (PMC5839573; doi:10.1371/journal.pone.0193993)
Supplement: S6 Appendix — (DOCX) [file pone.0193993.s006.docx]

**S6 Appendix:**

Example open-answers from teacher project evaluation questionnaire

**Which aspects of the project did you like most? / Comments on student’s enjoyment of project and benefits gained.**

“*A lovely aspect, which we were not expecting, was the children commenting on the environmental changes they observed over the six weeks; e.g. how many more leaves were on the trees – they generally became much more observant about the changes occurring within their school grounds*.”

*“That the resources were provided to us meaning that there was no extra work for teachers and that all the resources provided where child friendly and easy to follow.”*

*“The children enjoyed taking part in the bird surveys and checking on equipment. They liked taking the role of re-filling the birdfeeders etc. They loved spotting new birds and trying to identify them.”*

*“Children really enjoyed this project and went bird spotting every day. They were keen to share their experience with the rest of the school and so led an assembly explaining what they were doing – from that point on children in all classes were coming to tell us about birds they had spotted both at school and outside; even parents were coming and talking about how enthusiastic their children now were and many had been out to buy bird feeders. Towards the end of the project was very exciting as the feeders attracted other wildlife too – squirrels were seen each morning on the feeders within the first 4 weeks but during the last two weeks 1 feeder was constantly on the ground in the morning. As we were unsure why this was we fitted a camera outside to discover that we had a badger in the school grounds! They were also interested in the type of food that was preferred – suet blocks were most popular followed by sunflower seeds. By the end of the project the children were very confident with recognising the birds and are keen to try and learn how to identify more through their song.”*

*“Before the project, the enthusiasm was mixed throughout the class but during the project, every single child in the class has been fully engaged. Children come into school on a Monday morning discussing which birds they saw over the weekend and which bird calls/songs they may have heard. They have decided to run this project again after half term but try it in another area of the school. They are also going to support another class by teaching them how to identify the birds and show them how the project works – passing on all they have learnt. Many of them chose to play the games during wet break and lunch times.”*

*“High level of engagement. Most knew very little about different bird species and most now could name at least 4-5 different birds correctly. They loved the opportunity to be out of class, sitting quietly and watching ‘nature’.”*

“*Project allowed children allocated times for them to go out and observe what nature surrounds the school. Our school is quite concrete-based and the children do not automatically associate our school with nature. This has given the children time to reflect on this*”.

*“The children thoroughly enjoyed the project and were really enthusiastic about bird watching and wanting to make sure the food was kept topped up and the feeders remained clean. They learnt a lot about different species of bird and how they could be identified. They began to think about the habitats and what food attracted what birds. They want to continue to bird feed and are passionate about making sure the birds in our school are looked after”*

**What do you see as the long-term benefits of the project to the school/children?**

*“The school has become more aware of the wildlife in particular birds that inhabit the school and its surrounding grounds. As the teacher who ran the project, I would like to continue with the project to help develop the children’s knowledge as it makes a great link to the science curriculum.”*

*“Children wish to continue with the project and other children are wanting to participate. We are going to think of a way where we can have observation sheets readily available for children to complete. The class and other students wish to put feeders in other areas of the school grounds and see whether different species are attracted to different parts; they are also interested to find out whether the season will make a difference – they think more birds will visit the feeders in the winter as there isn’t as much natural food around. We want to put more bird boxes up in the grounds and have at least one with a camera fitted. The children (and staff) are very interested to see what other creatures visit the school ground. We have just planted a wild flower garden and would like to develop a wooded area and perhaps think about a pond in the future”.*

*“The children have shown a greater knowledge and awareness of the birds that surround the area. They now have a greater appreciation and respect towards the birds”.*

“*Many of the children have been inspired to bird watch and feed at home to ensure different species can flourish*”
